# Natural Hybrid Origin of the Controversial “Species” *Clematis* × *pinnata* (Ranunculaceae) Based on Multidisciplinary Evidence

**DOI:** 10.3389/fpls.2021.745988

**Published:** 2021-10-12

**Authors:** Rudan Lyu, Jian He, Yike Luo, Lele Lin, Min Yao, Jin Cheng, Lei Xie, Linying Pei, Shuangxi Yan, Liangqian Li

**Affiliations:** ^1^School of Ecology and Nature Conservation, Beijing Forestry University, Beijing, China; ^2^College of Biological Sciences and Technology, Beijing Forestry University, Beijing, China; ^3^Beijing Engineering Research Center for Landscape Plant, Beijing Forestry University Forest Science Co. Ltd., Beijing, China; ^4^College of Landscape Architecture and Art, Henan Agricultural University, Zhengzhou, China; ^5^Institute of Botany, The Chinese Academy of Sciences, Beijing, China

**Keywords:** *Clematis*, homoploid hybridization, morphology, niche modeling, phylogenomic analysis, species status

## Abstract

Interspecific hybridization is common and has often been viewed as a driving force of plant diversity. However, it raises taxonomic problems and thus impacts biodiversity estimation and biological conservation. Although previous molecular phylogenetic studies suggested that interspecific hybridization may be rather common in *Clematis*, and artificial hybridization has been widely applied to produce new *Clematis* cultivars for nearly two centuries, the issue of natural hybridization of *Clematis* has never been addressed in detail. In this study, we tested the hybrid origin of a mesophytic and cold-adapted vine species, *Clematis pinnata*, which is a rare and taxonomically controversial taxon endemic to northern China. Using field investigations, flow cytometry (FCM), phylogenomic analysis, morphological statistics, and niche modeling, we tested hybrid origin and species status of *C. pinnata*. The FCM results showed that all the tested species were homoploid (2n = 16). Phylonet and HyDe analyses based on transcriptome data showed the hybrid origins of *C.* × *pinnata* from either *C. brevicaudata* × *C. heracleifolia* or *C. brevicaudata* × *C. tubulosa*. The plastome phylogeny depicted that *C.* × *pinnata* in different sampling sites originated by different hybridization events. Morphological analysis showed intermediacy of *C.* × *pinnata* between its putative parental species in many qualitative and quantitative characters. Niche modeling results suggested that *C.* × *pinnata* had not been adapted to a novel ecological niche independent of its putative parents. These findings demonstrated that plants of *C.* × *pinnata* did not formed a self-evolved clade and should not be treated as a species. The present study also suggests that interspecific hybridization is a common mechanism in *Clematis* to generate diversity and variation, and it may play an important role in the evolution and diversification of this genus. Our study implies that morphological diversity caused by natural hybridization may overstate the real species diversity in *Clematis*.

## Introduction

Natural hybridization between species is a long-lasting topic in evolutionary biology (Mallet, 2007; Soltis and Soltis, 2009; Yakimowski and Rieseberg, 2014; Goulet et al., 2017). It is well acknowledged that hybridization plays an important role promoting the diversification of plants (Soltis and Soltis, 2009). About 35% of vascular plant species were estimated to be the results of interbreeding of different species (Wood et al., 2009). By now, hybridization is recognized as an important evolutionary force and a remarkable portion of speciation, and study the process of hybridization can aid us to understand the origin of new adaptations and plant diversity (Li et al., 2017). However, natural hybridization presents great challenges for taxonomy, biodiversity estimation, and conservation because a reticulated phylogeny is forced into a hierarchical taxonomic system (Zhang et al., 2020; Draper et al., 2021).

Interbreeding of different species is only the first step for hybrid speciation (Zhang et al., 2020), and not all natural hybridizations finally produce new species. Hybridization may finally result in a new species only if the hybrid lineage could be established as viable progenies through vegetative (or clonal) propagation, or allopolyploidy events, or other homoploid speciation mechanisms (Comai, 2005; Sochor et al., 2015; White et al., 2018). However, studies have shown that many previously recognized plant species represented F1 hybrids, which cannot be accepted as a real species (Zha et al., 2010; Zhang et al., 2020; Li et al., 2017). Because F1 hybrids tend to have similar morphologies due to the complete combination of parental genomes, they have often been recognized as distinct species by taxonomists (Liao et al., 2021). This raises critical problems for morphological-based taxonomy, biodiversity estimations and biological conservation. Interspecific hybridization also have many evolutionary consequences, including the origin and transfer of adaptations, the blur of distinctive lineages, or the formation of maladaptive hybrids, that have great impact on biodiversity conservation (Draper et al., 2021). For example, *Rosa pseudobanksiae*, previous recognized as an endangered species (category: CR), has recently been tested to be mostly F1 hybrids (Zhang et al., 2020). For this reason, it is critical not only to clarify whether a plant has hybrid origins, but also to know whether the taxon holds species status or not.

*Clematis* L. (Ranunculaceae) is a horticulturally important genus in the buttercup family (Ranunculaceae) with a world-wide distribution. Plants of *Clematis* are herbaceous or woody vines and, rarely, erect shrubs, or perennial herbs (Tamura, 1987, 1995; Wang and Li, 2005). *Clematis* species are mostly diploid (2n = 16), with only a few polyploid species (Tamura, 1995). Taxonomy of *Clematis* has been considered to be very challenging. There are great differences in species estimation of this genus, ranging from 240 (Tamura, 1995), 297 (Grey-Wilson, 2000), 320 (Johnson, 1997), to 354 (Wang and Li, 2005).

Recent molecular phylogenetic analyses showed extensive incongruence between nuclear and organellar phylogenies, suggesting that natural hybridization may be common among *Clematis* species (Miikeda et al., 2006; Xie et al., 2011; He et al., 2021). A recent molecular dating analyses based on complete plastid genome sequences (He et al., 2021) showed that *Clematis* diverged from its sister genus *Anemoclema* in early Miocene and its major clades evolved in the end of Miocene and early Pliocene. Species diversification of *Clematis* was estimated to be rather late mainly in Quaternary era, indicating recent species radiation in this genus (Xie et al., 2011; He et al., 2021).

The importance of hybridization in *Clematis* has been well known to horticulturists. Artificial hybridization can generate novel genotypic and phenotypic variants as well as novel ecological adaptations of great horticultural value, and has been widely applied in *Clematis* cultivar breeding for almost two centuries (Johnson, 1997; Toomey and Leeds, 2001). Artificial crossing between closely related *Clematis* species, or even between morphologically diverged taxa, can be easily done to produce various cultivars. By now, hundreds of *Clematis* cultivars have been produced through hybridization (Toomey and Leeds, 2001). For natural *Clematis*, recurrent hybridization events may be one of the key factors causing a great deal of trouble in its taxonomy. On the other hand, *Clematis* may also provide a new study systems to investigate the role and effects of hybridization on its global diversification. However, natural hybridization has never been addressed in detail for this genus.

**FIGURE 1:**
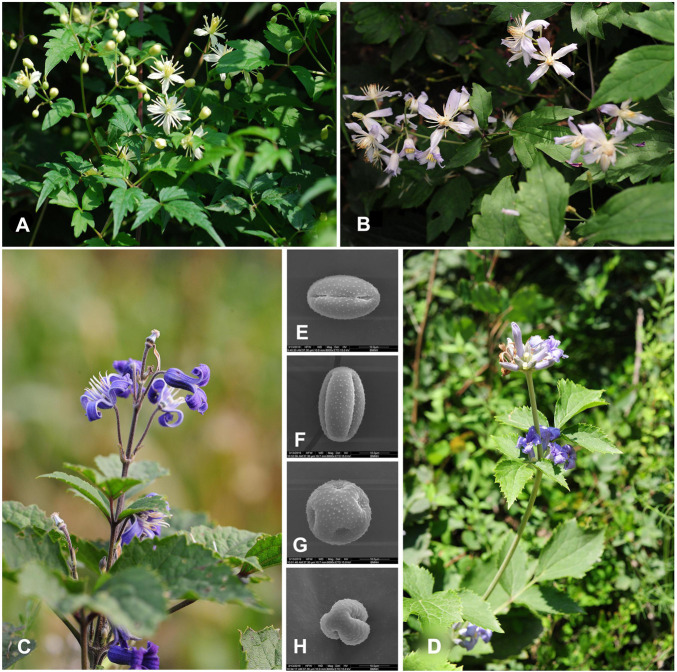
Pictures of *Clematis pinnata* and its putative parents and their representative pollen grains under an electron microscope. *C. brevicaudata* **(A)** and its pollen **(F)**; *C. pinnata* **(B)** and its pollen **(E)**; *C. heracleifolia* **(C)** and its pollen **(H)**; *C. tubulosa* **(D)** and its pollen **(G)**.

*Clematis pinnata* Maxim. is considered to be a rare and narrowly distributed species in Beijing and adjacent areas of northern China. Plants of *C. pinnata* are mesophytic, light-loving (when mature) and cold-adapted creeping vines. Taxonomic opinions differ (Xie et al., 2005; Wang and Xie, 2007) with some authors placing *C. pinnata* into sect. *Clematis* (Maximowicz, 1877; Handel-Mazzetti, 1939; Fang, 1980; Ting, 1980; Johnson, 1997), while others grouped it into sect. *Tubulosae* (Kuntze, 1885; Wang, 2001; Wang and Xie, 2007). In fact, *C. pinnata* shows morphological intermediacy between *C. heracleifolia* DC. (sect. *Tubulosae*) and *C. brevicaudata* DC. (sect. *Clematis*) or *C. tubulosa* Turcz. (sect. *Tubulosae*) and *C. brevicaudata* (Table 1 and Figure 1).

**TABLE 1:** A comparison of morphological characters of *Clematis pinnata* and its putative progenitors.

Characters	*Clematis pinnata*	*C. brevicaudata*	*C. heracleifolia/C. tubulosa*
Habit	Creeping or rarely climbing	Climbing	Erect herb sometimes with woody basal stem
Leaf	Pinnate, rarely bi-ternate and ternate	Bi-ternate, rarely pinnate	Ternate
Sepal color	Bluish white	Creamy white	Blue, purple
Calyces	From erect to spreading	Spreading	Erect
Filaments	With very inconspicuous hairs	Glabrous	Hairy
Pollen types	Tricolpate, often abnormal	Tricolpate	Tricolpate/pantoporate
Taxonomic treatment	Sect. *Clematis* (Ting, 1980) Sect. *Tubulosae* (Wang and Xie, 2007)	Sect. *Clematis* (Wang and Li, 2005)	Sect. *Tubulosae* (Wang and Xie, 2007)
Ploidy	Diploid	Diploid	Diploid
Distribution	Beijing, Hebei, Tianjin, Liaoning province of China	Northern, northeastern to southwestern China, N. Korea, Far East Russia	Northern, northeastern China to Korea

*Clematis heracleifolia* and *C. tubulosa* have very similar morphological characters and were often treated as the same species under the name *C. heracleifolia* (Rehder and Wilson, 1913; Handel-Mazzetti, 1939; Ting, 1980). However, Wang and Xie (2007) argued that *C. heracleifolia* and *C. tubulosa* can be distinguished from one another by their pedicels, sepal shapes, and pollen types (Xie and Li, 2012; Figure 1). So, they separated them into two different species. Our field survey showed that although in a wider geographical context, *C. heracleifolia* and *C. tubulosa* share a large, overlapping distribution range, their populations are often well separated from each other. Therefore, we treated them as two species, and when referring to them as one of the putative parent species we use *C. heracleifolia*/*C. tubulosa* hereafter.

Like *C. pinnata*, *C. brevicaudata*, and *C. heracleifolia*/ *C. tubulosa* are also mesophytic and cold-adapted species, but with much wider distribution ranges in China (Fang, 1980; Ting, 1980). However, their morphological features are quite different (Table 1). *Clematis brevicaudata* is climbing, and *C. heracleifolia*/*C. tubulosa* is erect perennial herb. Whereas, plants of *C. pinnata* are long and creeping but often not climbing. *Clematis pinnata*’s leaves are usually pinnate with five leaflets, albeit ternate or bi-ternate leaves are occasionally present on the upper or basal parts of individuals (Xie et al., 2005). In contrast, *C. brevicaudata* often carries bi-ternate leaves and *C. heracleifolia*/*C. tubulosa* always bears ternate leaves. The young flowers of *C. pinnata* are bluish-white and bell-shaped (similar to *C. heracleifolia*/*C. tubulosa*). However, when the flower is in full bloom, the sepals spread and fade to become almost white. At this stage, *C. pinnata* was often misidentified as *C. brevicaudata*. *Clematis pinnata*’s stamens are nearly glabrous and have inconspicuous hairs (Wang, 2001), which was also a misleading character in previous taxonomic treatments (Fang, 1980; Ting, 1980; Wang and Xie, 2007). Fore the four taxa, chromosome number of *C. brevicaudata* and *C. heracleifolia* have been reported to be diploid (2n = 2x = 16) (Gong et al., 1985; Zhang and He, 1990; Chung et al., 2013; Wang et al., 2017).

Despite their great morphological differences, *Clematis* sect. *Clematis* and sect. *Tubulosae* were shown to be very closely related by molecular phylogenetic studies (Miikeda et al., 2006; Xie et al., 2011; Lehtonen et al., 2016; He et al., 2021), thus indicating hybrid possibility between them. A Japanese species, *C. takedana*, has been considered as a natural hybrid species between *C. apiifolia* (sect. *Clematis*, distributed in Japan and eastern China) and *C. stans* (sect. *Tubulosae*, endemic to Japan) since its publication (Makino, 1907), solely based on its intermediate morphological characters between the latter two coexisting species. This case is very similar to *C. pinnata. Clematis brevicaudata* and *C. heracleifolia/C. tubulosa* always coexist in the present site of the morphologically intermediate *C. pinnata*. Furthermore, all of the four taxa have the same flowering time in late July to early September. High proportion of both aborted pollen and achene of *C. pinnata* have been reported by Shi (2003). She also assumed that *C. pinnata* may be a hybrid taxon between *C. brevicaudata* and *C. heracleifolia*. However, her allozyme, ITS, and *trn*L-F analyses did not give sufficient results for testing this assumption. From all the above-mentioned evidence, we hypothesize that *C. pinnata* is a homoploid hybrid between *C. brevicaudata* and *C. heracleifolia*/*C. tubulosa*, and its species status needs to be tested.

When testing the hybrid origin of a plant, a clearly defined species concept must be adopted. Although morphological-based species concept has been widely used in plants because of its practicability for taxonomic purposes (Soltis and Soltis, 2009), it cannot be applied for testing hybridization hypothesis. The biological species concept, which emphasizes reproductive isolation, has played a major role in views of plant taxa (Soltis and Soltis, 2009). However, when testing hybrid hypothesis, this species concept is not operationally useful for empiricists (Feliner et al., 2017). In this study, we adopted broader conceptual frameworks for the definition of homoploid hybrid speciation in accordance with those proposed by Mallet (2007), Abbott et al. (2013), and Feliner et al. (2017). A homoploid hybrid species should have a clear genetic evidence of hybridization without polyploidization, give rise to an established self-evolving lineage, and be morphologically and ecologically distinct (especially in view that *C. pinnata* has the same phenological period with the putative parents) from its progenitors.

Therefore, using a multidisciplinary data including flow cytometry, phylogenomics, morphological statistics, and ecological niche modeling analysis, we test the possible homoploid hybrid origin and species status of *C. pinnata*. We focus on questions about the origin and process of the homoploid hybridization of *C. pinnata*. Flow cytometry determines ploidy level of *C. pinnata* compared to the related species. PhyloNet and HyDe analyses using transcriptome data give genetic evidence of hybridization, whereas the complete plastome analyses examine the maternal inheritance of *C. pinnata*. Using morphological statistics, we assess whether *C. pinnata* is intermediate between its putative parents in quantitative characters. We also model the niche overlap between *C. pinnata* and its putative parental species to test whether *C. pinnata* acquired new ecological niche independent of its parents. The aims of this study are (1) to test whether *C. pinnata* is of homoploid hybrid origin, (2) to determine which species are involved in hybridization events, (3) to determine the direction of hybridization, and (4) to test species status of *C. pinnata*.

## Materials and Methods

### Study Locations and Field Investigation

Distribution range of *C. pinnata* is concentrated in Beijing and adjacent areas in Tianjin and Hebei Province, only with a few records in Liaoning Province (explained in Taxonomic Treatment). From 2017 to 2019, we surveyed *C. pinnata* over its entire distribution range and found multiple distribution sites in Beijing and Liaoning Province (Table 1). In all its distribution sites, both of its putative parental species are present (Table 2 and Supplementary Figure S1). We recorded GPS coordinates and altitude of each population, and collected leaf tissues as well as specimens for flow cytometry experiments, phylogenomic studies, and morphological analyses. Individuals of *C. pinnata* are rare, often with a few plants in each site. So, we collected all the individuals in each site. In total, we gathered 27 *C. pinnata* individuals and one dubious plant from eight collecting sites in Beijing and Liaoning Province. A dubious individual was collected in Baihuashan (BHS), Beijing. It was not in flower when collected. The plant was creeping with pinnate leaves, which was similar to *C. pinnata*. However, the leaflets were smaller than typical *C. pinnata* and very similar to those of *C. brevicaudata*. We included this dubious individual in our phylogenomic study to determine its identity.

**TABLE 2:** Locations of the population sites of *Clematis pinnata* and its putative progenitors collected in this study.

Locality and Pop. ID	Taxon	Collector	Voucher number	Number of specimens collected	GPS coordinates	Elevation (m)
Sizuolou conservation area, Pinggu, Beijing (SZL)	*Clematis brevicaudata*	*R.D. Lyu*	*LRD0133*	2	E: 117.2591° N: 40.3259°	780
	*C. tubulosa*		*LRD0058*	5		
	*C. pinnata*		*LRD0053*	5		
Laoquan mountain park, Pinggu, Beijing (LQ)	*C. brevicaudata*	*R.D. Lyu*	*LRD0024*	6	E: 117.1275° N: 40.3098°	300
	*C. heracleifolia*		*LRD0009*	10		
	*C. pinnata*		*LRD0026*	1		
Baihuashan, Mentougou, Beijing (BHS)	*C. brevicaudata*	*R.D. Lyu*	*20190821-01*	5	E: 115.5789° N: 39.8393°	1155
	*C. tubulosa*		*20190821-02*	5		
	*C. pinnata* ?		*20190821-03*	1		
Jiufeng forest park, Haidian, Beijing (JF)	*C. brevicaudata*	*R.D. Lyu*	*20180511-01*	8	E: 116.0764° N: 40.0598°	720
	*C. tubulosa*		*LRD0002*	15		
	*C. pinnata*		*LRD0008*	2		
Yunmengshan, Huairou, Beijing (YMS)	*C. brevicaudata*	*R.D. Lyu*	*LRD0084*	11	E: 116.6849° N: 40.5839°	1120
	*C. tubulosa*		*LRD0085*	22		
	*C. pinnata*		*LRD0083*	1		
Woguayu village, Pinggu, Beijing (WGY)	*C. brevicaudata*	*R.D. Lyu*	*LRD0132*	6	E: 117.1276° N: 40.2560°	320
	*C. heracleifolia*		*LRD0070*	15		
	*C. pinnata*		*LRD0068*	2		
Sanyang ancient volcano, Pinggu, Beijing (SYG)	*C. brevicaudata*	*R.D. Lyu*	*LRD0131*	19	E: 117.1342° N: 40.2850°	280
	*C. heracleifolia*		*LRD0039*	29		
	*C. pinnata*		*LRD0033*	6		
Dongling park, Shenyang, Liaoning province (SY)	*C. brevicaudata*	*R.D. Lyu*	*LRD0110*	37	E: 123.5854° N: 41.8366°	90
	*C. tubulosa*		*LRD0106*	36		
	*C. pinnata*		*LRD0105*	10		
Jianchuan, Dali, Yunnan	*Anemoclema glaucifolium*	*L. Xie*	*20190715-04*	1	E: 99.9031° N: 26.5434°	2240
Nanshiyang valley, Mentougou, Beijing	*Clematis acerifolia*	*M. Yao*	*YM004*	1	E: 115.7190° N: 40.0922°	380
Jiufeng forest park, Haidian, Beijing	*C. hexapetala*	*L. Xie*	*JF-6*	1	E: 116.0764° N: 40.0598°	688
Datong, Shanxi	*C. fruticosa*	*J. He*	*20170064*	1	E: 113.5466° N: 39.8403°	1194
Baojiakou, Zhuolu, Hebei	*C. intricata*	*L. Xie*	*2019052001*	1	E: 115.3682° N: 40.0872°	1141
Xiaowutai Mt., Yuxian, Hebei	*C. ochotensis*	*L. Xie*	*2019051801*	1	E: 115.0614° N: 40.0026°	1423

*Collecting information of the outgroups is also presented. Vouchers are deposited in the Herbarium of Beijing Forestry University (BJFC).*

*Pop. ID, Population ID; ?, The identity of this sample is dubious.*

From all the eight collecting sites, we gathered 94 individuals of *C. brevicaudata*. We also collected 91 individuals of *C. tubulosa* from five of the eight sites, and 49 individuals of *C. heracleifolia* from the rest of the three sites. The individuals were randomly collected in each site, and the distance between individuals is more than 20 meters. Furthermore, five other *Clematis* species (*C. hexapetala* Pall., *C. acerifolia* Maxim., *C. ochotensis* (Pall.) Poir, *C. intricata* Bunge, and *C. fruticosa* Turcz.) commonly found in northern China were also included in the phylogenomic analysis as outgroups. Voucher specimens were deposited in the Herbarium of Beijing Forestry University (BJFC, Supplementary Table S1).

### Ploidy Level Detection

We used a flow cytometry (FCM) method (Doležel et al., 2007; Bourge et al., 2018) to determine whether *C. pinnata* is homoploid with its putative parents. We used samples from Jiufeng forest park to check if *C. pinnata* is homoploid with the putative parents in the same site. Because only *C. brevicaudata* and *C. tubulosa* are present in Jiufeng population, we checked *C. heracleifolia* using samples from Yanqing district.

The FCM measurement was carried out in the Key Laboratory of Photobiology of Institute of Botany, the Chinese Academy of Sciences. Preparation of dried leaf samples of the four taxa basically followed a standard two-step method to isolate plant cell nuclei (Qu et al., 2018). For each sample, about 20–30 mg of dried leaf was chopped in 1 mL of LB01 lysis buffer (15 mM Tris, 2 mM EDTA-Na_2_, 0.5 mM spermine tetrahydrochloride, 80 mM KCl, 20 mM NaCl, 0.1% Triton X-100, 15 mM β-mercaptoethanol, pH 7.5) to release cell contents. The resulting culture was gently pipetted and filtered through a 400-mesh screen to remove cell debris. The samples were then stained with 50 μL 1 mg⋅mL^–1^ propidium iodide (PI) and 50 μL 1 mg⋅mL^–1^ RNase A in an ice bath for 10 min before being analyzed using a MoFlo XDP flow cytometer (Beckman Coulter Inc., United States). We measured about 5000 nuclei from each sample (individual) and performed three replicates (three individuals) for each species. PI conjugated to Alexa Fluor 488 (Molecular Probes) was excited with a 488-nm argon-ion laser and the fluorescence was detected using a microscope equipped with a 625/26-nm HQ bandpass filter. Mean channel positions and coefficients of variation (CVs) of the G0/G1 peaks were calculated using Summit 5.2 software (Beckman Coulter Inc., United States). We used *C. brevicaudata* as the external standard reference for our flow cytometry measurements.

### Transcriptome Analysis

To examine the possible hybrid origin of *C. pinnata*, we generated transcriptomes from samples of the eight collecting sites. Fresh leaf materials of the *Clematis* samples were collected from field and quickly deposited in liquid nitrogen. For *C. pinnata*, leaf tissues of all the individuals (from 1 to 10 individuals per site, Table 2) in each site were mixed together, respectively. Population transcriptome data of *C. brevicaudata* and *C. heracleifolia*/*C. tubulosa* were represented by five individuals for each species in each site. Five other *Clematis* species (*C. ochotensis*, *C. intricata*, *C. hexapetala*, *C. fruticosa*, and *C. acerifolia*, one individual for each species) were included for transcriptomes analysis, and *Anemoclema glaucifolium* was chosen as the outgroup.

We extracted total RNA from sampled leaves by using TRIzol Reagent (Invitrogen, Thermo Fisher Scientific, Shanghai, China) and sent the RNA samples to Biomarker Technologies^1^ for cDNA library preparation and Illumina 2 × 150 bp paired-end sequencing. About 6 Gb of raw reads were obtained for each sampled species. The raw reads for the 30 newly generated transcriptomes were deposited in the NCBI Sequence Read Archive under BioProject PRJNA657443 (Supplementary Table S1). Then, they were filtered and trimmed using Trimmomatic v. 0.39 (Bolger et al., 2014). The clean RNA-Seq reads were *de novo* assembled using Trinity v. 2.5.1 (Grabherr et al., 2011) with default parameters. Because the assembly had a large number of redundant transcripts, we kept the longest isoforms of the related contigs by using the “get_longest_isoform_seq_per_trinity_gene.pl” utility in Trinity to let each unigene represent a collection of expressed sequences that apparently came from the same transcription locus. We used CD-HIT (Li and Godzik, 2006; Fu et al., 2012) to remove redundant sequences from the unigene and TransDecoder v 5.0 to predict protein-coding regions (Haas et al., 2013).

Single-copy orthologous nuclear genes (SCOGs) can be determined and screened out by reference-based methods (Zhao et al., 2013; Zeng et al., 2014, 2017; Feng et al., 2019) and all-against-all alignment methods without references (Sveinsson et al., 2014; Nishikawa et al., 2015; Majeed et al., 2019). The second strategy takes much more computational time, but can find more complete single-copy orthologous dataset than the former strategy, especially when there are no close genomic references available (Nishikawa et al., 2015). In this study, our method followed that of Nishikawa et al. (2015), which was based on all-against-all alignment with BLASTP as implemented in Proteinortho v. 6 (Lechner et al., 2011). The definition of an orthologous relationship between proteins was: *e* ≤ 1 × 10^5^, identity ≥ 25%, and alignment coverage ≥ 50%. The extracted SCOGs were aligned using MAFFT (Katoh et al., 2005), and each alignment was processed with a Python script^2^ to remove ambiguous aligned positions.

For the phylogenetic analysis of the SCOGs data, both coalescent- and concatenation-based methods were applied. The coalescent-based method was conducted by the Accurate Species Tree Algorithm v. 4.4.4 (ASTRAL; Mirarab et al., 2014). Single-gene ML tree was reconstructed in RAxML v. 8.1.17 (Stamatakis, 2014) under the GTR + G model as suggested in the manual. Then, we used TreeShrink (Mai and Mirarab, 2018) to test whether long branch attraction caused incorrect phylogenetic relationship and then deleted all the trees with suspicious patterns of branch length. All the remaining ML trees were then used as input trees for ASTRAL. For the concatenation method, the aligned SCOGs sequences were linked using Geneious Prime 2019 (Kearse et al., 2012). Then, we used a ML method for phylogeny reconstruction as described above. In view that the presence of hybrid taxa may cause problems in phylogenetic inference (Xu, 2000), we exclude putative hybrid taxa (*C. pinnata* and *C. ochotensis* inferred by Phylonet analysis, see below) and re-do the above mentioned phylogenetic analysis to confirm the backbone of *Clematis* phylogeny.

Because complex evolutionary scenarios such as hybridization events do not proceed in tree-like manners (Huson and Bryant, 2006), we used the NeighborNet method implemented in SplitsTree 4.11.3 (Huson and Bryant, 2006) to reconstruct phylogenetic networks for the concatenated dataset. We excluded insertions/deletions (indels) and used the K2P model (Kimura, 1980) for distance analysis and support values at each node were estimated by running 1000 bootstrap replicates.

Then, we analyzed potential hybridization of *C. pinnata* using pseudolikelihood approach with PhyloNet (Wen et al., 2018), a software that detects phylogenetic networks based on the multispecies coalescent model, to directly infer the hybridization process. Because short gene alignments could cause random errors in phylogenetic inference in PhyloNet analysis (McLean et al., 2019), we selected gene alignments that were at least 500 bp in length for analysis. We divided SCOGs alignments into eight population datasets because PhyloNet cannot test too many hybridization events in a phylogenetic tree due to computational restrictions (Wen et al., 2018; Morales-Briones et al., 2021). Each dataset contains nine taxa: *C. pinnata*, both its putative parents, five other *Clematis* species, and *Anemoclema* as the outgroup. All the separate SCOGs phylogenies were inferred using an ML method as described above. We adopted the maximum pseudolikelihood method to model incomplete lineage sorting and gene flow using individual gene trees with the command InferNetwork_MPL (Yu and Nakhleh, 2015). Each Network search was allowed 0–4 reticulations and the log likelihood score for each network was also inferred. The best number of hybridization events was selected by plotting the likelihood scores. A sharp likelihood score increase is expected until it reaches the best number, after which the score increases slowly. For the dubious individual in BHS population, we also conducted another PhyloNet analysis by excluding another putative hybrid taxa *C. ochotensis* for comparison.

To further verify hybrid origin of *C. pinnata* and possible gene flow between the putative progenitors, we used an alternative method similar to D-statistic (or ABBA-BABA tests) analysis (Patterson et al., 2012) for comparing the results with the PhyloNet analysis. In this study, we used HyDe v. 0.4.1a (Blischak et al., 2018) to check for hybridization and gene flow. This method considered both hybridization and coalescence in a unified framework and can be used to fast detect both current and ancient hybridization events using genomic SNP data (Kubatko and Chifman, 2019). It assesses statistical significance of hybridization by testing for hybridization among all possible triplet combinations of the sampled species. Since HyDe uses single nucleotide polymorphism (SNP) data, the SCOG alignments were concatenated to construct a super SNPs (complete SNP data, hereafter) matrix using Phyutility v. 2.2 (Smith and Dunn, 2008) with removing the missing sites. We then used HyDe with a Python script, run_hyde.py (Blischak et al., 2018) to test possible hybrid origin of *C. pinnata* from each of the eight collection sites. The software assumed that each one of the three tested species (*C. pinnata*, *C. brevicaudata*, *C. heracleifolia/C. tubulosa*) is of a hybrid origin of the other two species. Thus, in total, 169 possible hybrid triplet combinations in each collecting site were tested.

Linkage disequilibrium (LD) may bias the results when analyzing genomic SNP data (Nielsen and Signorovitch, 2003; Malomane et al., 2018). Studies have shown that using LD-pruned data can effectively correct such biases (Hoeffding et al., 2017; Malomane et al., 2018; Davenport et al., 2020). However, accurately pruning LD sites from total SNP dataset needs high quality whole genomic reference (at the chromosome level). Because *Clematis* has huge genome size with the reported mean 1C value of 10.48 pg (Min: 6.90 pg and Max: 15.80 pg^3^), there are still no whole genome references published to date. For this reason, we used another approach to relieve the influence of LD. We randomly selected one SNP from each SCOG alignment using a python script^4^ to obtain a reduced SNP dataset, and then the above mentioned HyDe analysis was also conducted using this dataset.

A Z-statistical test was conducted and probabilities of genetic contributions of putative parents (γ and 1–γ) were calculated. According to Meng and Kubatko’s (2009) hybrid model, the hybrid taxa is either sister to “P1” with probability (γ) or sister to “P2” with probability (1–γ), The null hypothesis was that when hybridization was absent, the expected value of γ should be 0.

### Chloroplast Genome Analysis

Because chloroplast (cp) genomes inherit maternally in Ranunculaceae (Corriveau and Coleman, 1988), we used genome skimming data to assemble complete cp genomes to determine the maternal parent of *C. pinnata*. For all the eight collecting sites, one individual of *C. pinnata* and its putative parental species was chosen for DNA extraction and library construction. Five other *Clematis* and one *Anemoclema* species (Zhang et al., 2015; Jiang et al., 2017) were also included in the analysis (Table 2).

We used genomic DNA extraction kits (Tiangen Biotech Co. Ltd., Beijing) to extract total genomic DNAs from silica-dried leaves. Extracted DNA was then sent to Biomarker Technologies (Beijing, China) for library construction and next-generation sequencing (NGS). Paired-end reads of 2 × 150 bp were generated on an Illumina Hiseq 4000 genome analyzer platform and raw reads were filtered using the FASTX-Toolkit^5^ to obtain high-quality data by deleting adaptors and low-quality reads.

We then used the Map to Reference option in Geneious Prime 2019 (Kearse et al., 2012) and reference sequences (MG675223.1 and MG675222) to filter out cp reads. Putative cp reads were used for *de novo* assembly using Geneious Prime 2019 (Kearse et al., 2012) with a low sensitivity setting to reconstruct the complete cp sequence. Gaps were bridged using 20 replicates in a FineTuning step in Geneious Prime 2019 (Kearse et al., 2012). When a contig containing a large single copy (LSC), a small single copy (SSC), and an inverted repeat (IR) region was assembled, the other IR region was determined and attached to the contig using the Repeat Finder function in Geneious Prime 2019 (Kearse et al., 2012) to construct the complete cp genome sequence. The assembled plastome sequences were annotated using Plastid Genome Annotator (Qu et al., 2019), and then checked manually in Geneious Prime 2019 (Kearse et al., 2012). We used the Organellar Genome DRAW tool to illustrate the newly sequenced cp genomes (Lohse et al., 2013) with accession numbers from MT796599 to MT706622 (Supplementary Table S2).

The complete cp genome dataset was aligned using MAFFT v 6.833 (Katoh et al., 2005). Ambiguous alignments and sites with more than 80% missing data were deleted automatically using a Python script^6^. We used the maximum likelihood (ML) and Bayesian inference (BI) methods for phylogeny reconstruction because these methods are less sensitive to long-branch attraction artifacts than the parsimony method (Bergsten, 2005). The ML trees were generated using RAxML v 8.1.17 (Stamatakis, 2014) under the GTR + G model, with bootstrap percentages computed after 100 replicates.

Bayesian inference was performed with MrBayes v3.2.3 (Ronquist and Huelsenbeck, 2003). The models were tested and set for BI analysis according to our previous study (He et al., 2019). Two Markov chain Monte Carlo (MCMC) chains were run independently. Each consisted of three hot chains and one cold chain for 5,000,000 generations. The trees were sampled every 100 generations. The convergence of the Markov chain was tested by calculating the standard deviation value of split frequencies (less than 0.01). The first 20% of trees were removed as burn-in. The remaining trees were used to construct the consensus tree.

### Morphological Analysis

Both hybrids and hybrid species are expected to fall in between parental progenitors in morphology (Zhang et al., 2020). To test hybridization hypothesis of *C. pinnata*, 16 qualitative and ten quantitative morphological characters were chosen for analysis (Supplementary Tables S3, S4). In total, 278 specimens of the four tested taxa (*C. pinnata*: 29; *C. brevicaudata*: 124, *C. heracleifolia*: 56, and *C. tubulosa*: 69) from multiple herbaria were measured (information of all the specimens is presented in Supplementary Table S5). The selected qualitative characters are taxonomically important traits based on previous taxonomic study (Wang and Xie, 2007). We measured more than 90 leaflets, stems, and flowers of each species to retrieve the quantitative characters.

For the 10 quantitative characters, Kolmogorov–Smirnov test and *F*-test were used for normality test. Normal distribution data was used one-way ANOVA (analysis of variance) to analysis, and non-normal distribution data was used Kruskal–Wallis test to analysis via IBM SPSS statistics software v25 (SPSS Inc., Chicago, IL, United States), to test the significant differences among the four tested species. Significant differences between any two taxa were identified using a *post hoc* Tukey’s honest significant difference (HSD) test with false discovery rate (FDR) correction (Benjamini and Hochberg, 1995). Box charts and principal component analysis (PCA) were applied to visualize the differences between all the taxonomic units by R (R Core Team, 2018).

### Ecological Niche Modeling

To determine whether *C. pinnata* has its own ecological niche independent of its parents, we used species distribution models (SDMs) with 278 distribution records (Supplementary Table S5) (*C. pinnata*: 29; *C. brevicaudata*: 124, *C. heracleifolia*: 56, and *C. tubulosa*: 69 from their entire distribution ranges) and 33 high-resolution environmental variables to project and predict the current distribution patterns of each taxon. The distribution records were obtained from our field investigation and the Chinese Virtual Herbarium database (CVH^7^). For CVH data, the identification of specimen was checked based on the previous taxonomic revision (Wang and Xie, 2007). When no exact GPS information was recorded for a specimen, the geographic coordinates were determined using Google Earth 7.0^8^. In order to avoid sampling bias, only one individual was retained in each 1.0 × 1.0 km square using the “spatially rarify occurrence data” tool in the SDM toolbox for the ArcGis software (Esri, Redlands, CA, United States).

Because many ecological factors, such as bioclimatic, sun light, vegetation, terrain, and soil, may influence the distribution of the four tested species, we obtained 33 environmental variables with 2.5′ spatial resolution (Supplementary Table S6) to conduct niche modeling. Those variables consist of 19 bioclimatic variables from WorldClim-Global Climate Data^9^ (Hijmans et al., 2005); six UV-B radiation variables from a global UV-B radiation dataset for macroecological studies^10^ (Beckmann et al., 2014); five vegetation and terrain variables from Harmonized World Soil Database v 1.2 of the Food and Agriculture Organization^11^ (Food and Agriculture Organization [FAO] and International Institute for Applied Systems Analysis [IIASA], 2012), and three soil variables from the University of Wisconsin^12^ (New et al., 1999).

Because collinearity between environmental variables can lead to wrong modeling results (Dormann et al., 2013), we applied the Pearson correlation coefficients using the “banding collection statistics” tool in ArcGis 10.2 and the Jackknife test implemented in MaxEnt to evaluate the contributions of each variable. All the environment variables were converted to ASCII format using an ArcGIS 10.2 conversion tool. Next, we obtained a map of Asia from the global administrative region database^13^. After removing overly correlated variables (*r* < 0.7) (Guisan and Thuiller, 2005), we performed predictive analysis for each species using MaxEnt 3.4.1^14^. We ran 10 bootstrap replicates, in which 25% was used for model testing and the other 75% of the presence data was randomly selected for model training to optimize the model. The model was parameterized with a maximum of 10,000 background points, a convergence threshold of 0.00001, and a maximum of 500 interactions (Lin et al., 2020).

We used area under the curve (AUC) values to evaluate model prediction accuracies. An AUC value is the area enclosed by the receiver operating characteristic curve and the abscissa, and the closer the value is to 1, the more predictively accurate the model is (Fielding and Bell, 1997; Babar et al., 2012). We used the jackknife method to evaluate the weight of each environmental factor on the distribution area, and based on these values, we classified potentially suitable habitats into four categories (which was widely applied by other niche modeling studies, such as Tang et al., 2017; Shitara et al., 2018; Lin et al., 2020, and many others) in a final predictive map of species: unsuitable (<0.2), barely suitable (0.2–0.4), moderately suitable (0.4–0.6), highly suitable (>0.6).

Then, the ecological niche overlap between tested species was checked following the methods by White et al. (2018). We tested niche overlap of species pairs using the ENMtools 1.4.4 (Warren et al., 2010) by calculating Schoener’s *D* (Schoener, 1968) and Warren’s *I* statistic values, which 0 indicates no overlap and 1 indicates full overlap (Warren et al., 2008). We then used a niche equivalence test species pairs by comparing statistics *D* and *I* using 100 pseudo-replicates (Warren et al., 2008) to test whether the species pairs have identical ecological niche modeling under the null hypothesis. When the statistic value is smaller than the pseudo-replicates value, the result indicates that the two species did not occupy the same ecological niche.

Next, to assess the extent of niche overlap between species pairs, we applied environmental PCA (PCA-env) method (Broennimann et al., 2012) as implemented in the R package of ecospat (Di Cola et al., 2017; R Core Team, 2018)^15^. We transformed the multidimensional space of environment variables into two-dimensional space by means of PCA. Following White et al. (2018), we set the resolution to 100 with each grid corresponding to a unique environmental space as suggested by Broennimann et al. (2012), and then we calculated the smoothness of species occurrence by using kernel density to project species onto a grid. We used Schoener’s *D* (Schoener, 1968) and Warren’s *I* statistics (Warren et al., 2008) to calculate niche overlap and we accepted the null hypothesis (two species’ niches were equivalent) when *p* < 0.05.

## Results

### Ploidy Level Detection

Our FCM results showed that the ratio of the mean (G_0_/G_1_) of *C.* × *pinnata*, *C. tubulosa*, and *C. heracleifolia* to that of the reference (*C. brevicaudata*) ranged between 0.95 and 1.22. These results showed that all tested species were of the same ploidy level, i.e., 2n = 16 (Supplementary Tables S7, S8 and Supplementary Figure S2).

### Transcriptome Data Analysis

**FIGURE 2:**
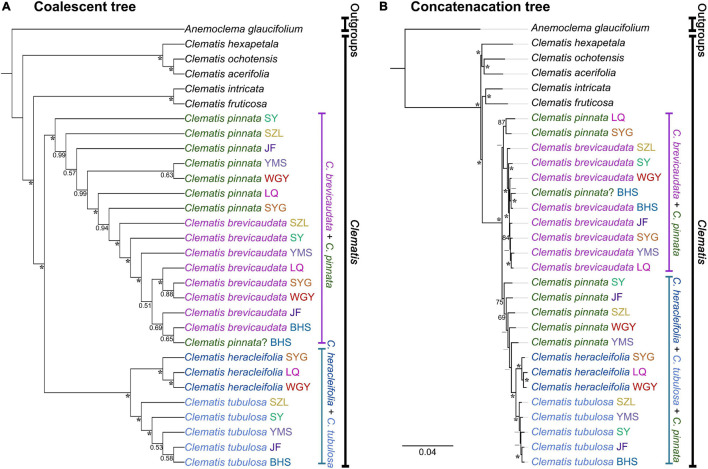
Phylogenetic relationships of eight populations of *Clematis pinnata* and its close relatives inferred by 3170 co-orthologous nuclear genes using concatenated and coalescent methods. **(A)** Concatenated phylogeny inferred using maximum likelihood method. Bootstrap percentages are indicated on the branches. * Shows that ML bootstrap values are 100, while – shows that support values are less than 50. **(B)** Coalescence based species tree, inferred by ASTRAL. Numbers at branches are local posterior probabilities. Local posterior probabilities with values equal to 1.00 were marked with * at branches. Population location name abbreviations are explained in Table 2.

**FIGURE 3:**
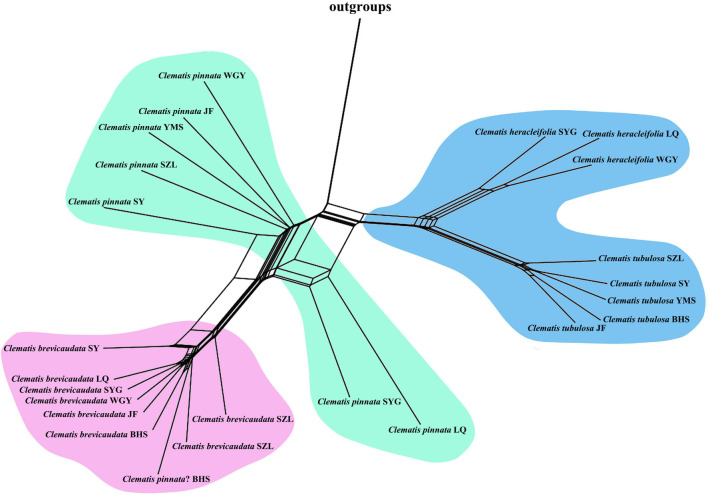
Molecular phylogenetic network among *Clematis pinnata* and its putative parents from co-orthologous nuclear gene dataset (3170 SCOGs). Distance analysis was ran under K2P model (Kimura, 1980) and support values were estimated by running 1000 bootstrap replicates.

We generated 30 transcriptome datasets from the four tested species and other outgroup species in this study. Detailed information of the transcriptome data was presented in Supplementary Table S1. Because the sampled species are closely related, we filtered out 3198 SCOGs without missing data, using all-against-all alignment strategy. Among them, 28 SCOGs were tested to generate bad trees with suspicious branch length by TreeShrink. So, we kept 3170 SCOGs for phylogenetic reconstruction. Our results showed that the phylogenetic backbone without putative hybrid taxa (*C.* × *pinnata* and *C. ochotensis*, Supplementary Figure S3) is fully consistent with the results from phylogenetic analysis with inclusion of the hybrid taxa (Figure 2). *Clematis* × *pinnata*, *C. brevicaudata*, *C. heracleifolia*, and *C. tubulosa* formed a well-supported clade by both concatenated (ML BS = 100) and coalescent methods (Local posterior probabilities, LPP = 1, Figure 2). However, the resolution and support values within this clade are low, and samples of *C.* × *pinnata* were not tested to be monophyletic within the clade. The network analysis (Figure 3) showed that *C. brevicaudata* and *C. heracleifolia/C. tubulosa* are well separated. *C. heracleifolia* and *C. tubulosa* are closely related but formed two strains, respectively. Samples of *C.* × *pinnata* did not form a single strain in this analysis.

**FIGURE 4:**
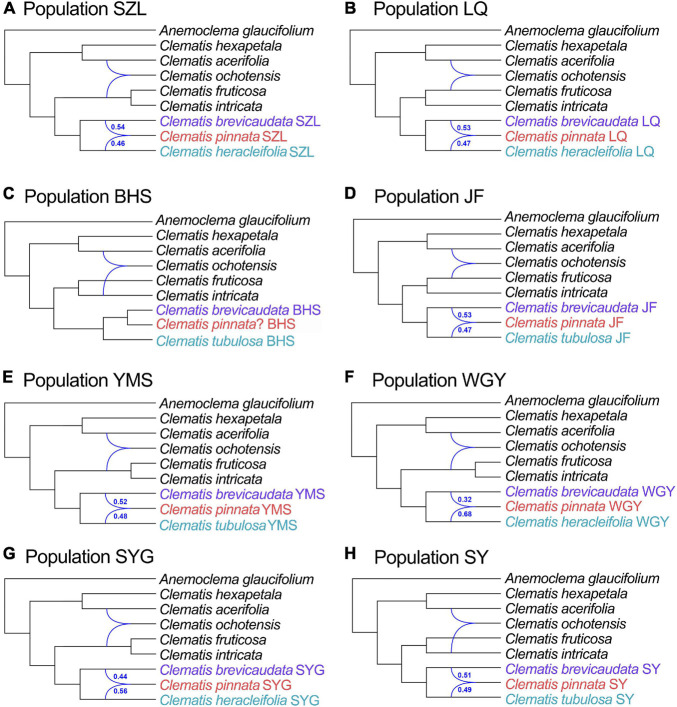
Optimal species networks of all eight populations (**A-H**, population abbreviations are explained in Table 2) of *C. pinnata* and its putative parents inferred by 3000 co-orthologous nuclear genes (at least 500 bp in length) using the PhyloNet. The results are maximum pseudolikelihood trees with a maximum of two reticulations. Genetic contributions of the putative parents estimated by HyDe analysis (using reduced SNP data) were marked beside the curve lines.

We selected 3000 SCOGs with aligned length longer than 500 bp for PhyloNet analysis. All the eight tested populations showed a gently increasing probability value when the maximum number of reticulations was set to two (Supplementary Figure S4). For this reason, we displayed and discussed the results based on the setting of two maximum hybridization events (Figure 4). The results showed that, except for the dubious sample in population BHS, samples of *C.* × *pinnata* from the other seven populations were tested to be hybrids between *C. brevicaudata* and *C. heracleifolia*/*C. tubulosa*. Another possible hybridization event occurred in *C. ochotensis* which belongs to *C.* sect. *Atragene* (Figure 4). For population BHS, the dubious sample was not tested to be a hybrid between *C. brevicaudata* and *C. tubulosa* but more closely related to *C. brevicaudata* in the PhyloNet analyses either with (Figure 4) or without *C. ochotensis* (Supplementary Figure S5).

In this study, we obtained 316,066 and 3170 SNPs for the complete and reduced SNP datasets, respectively, for HyDe analysis. In general, the two datasets generated similar results (Table 3 and Supplementary Table S9), and hybrid origin of *C.* × *pinnata* was confirmed by this analysis. The results from the reduced SNP data (as well as the complete SNP data) showed that the genetic contributions of the putative parents were nearly equal in six of the eight populations (Table 3). In WGY, the parental genetic contribution of *C. brevicaudata* was 0.33, while genetic contribution (1-γ) of *C. heracleifolia* was 0.67. For the dubious sample from population BHS, HyDe analysis for the complete SNP data detected weak possible hybrid origin of the dubious plant between *C. brevicaudata* and *C. tubulosa* (positive *z*-score = 3.74) with very low genetic contribution of *C. tubulosa* (1-γ = 0.01, Supplementary Table S9). However, analysis of the reduced SNP data did not support hybrid origin of the dubious plant (Table 3) which is consistent with the PhyloNet analysis.

**TABLE 3:** HyDe analysis of *Clematis pinnata* and its putative parents in each population using reduced SNP dataset.

Populations	Putative parents 1	Putative hybrid species	Putative parents 2	Z-score**	P-value	Γ ***
BHS*	*Clematis pinnata*?	*Clematis brevicaudata*	*Clematis tubulosa*	1.81	0.04	0.98
	*Clematis pinnata*?	*Clematis tubulosa*	*Clematis brevicaudata*	–107.41	1.00	0.50
	*Clematis brevicaudata*	*Clematis pinnata*?	*Clematis tubulosa*	–1.78	0.96	1.02
JF	*Clematis pinnata*	*Clematis brevicaudata*	*Clematis tubulosa*	–1.81	0.97	0.09
	*Clematis pinnata*	*Clematis tubulosa*	*Clematis brevicaudata*	–1.62	0.97	–0.13
	***Clematis brevicaudata***	***Clematis pinnata***	***Clematis tubulosa***	**15.56**	**0.00**	**0.47**
LQ	*Clematis pinnata*	*Clematis brevicaudata*	*Clematis heracleifolia*	–1.68	0.95	0.09
	*Clematis pinnata*	*Clematis heracleifolia*	*Clematis brevicaudata*	–1.51	0.93	–0.13
	***Clematis brevicaudata***	***Clematis pinnata***	***Clematis heracleifolia***	**14.78**	**0.00**	**0.47**
SY	*Clematis pinnata*	*Clematis brevicaudata*	*Clematis tubulosa*	–0.47	0.68	0.029
	*Clematis pinnata*	*Clematis tubulosa*	*Clematis brevicaudata*	–0.46	0.68	–0.03
	***Clematis brevicaudata***	***Clematis pinnata***	***Clematis tubulosa***	**15.29**	**0.00**	**0.49**
SYG	*Clematis pinnata*	*Clematis brevicaudata*	*Clematis heracleifolia*	–3.00	1.00	–0.37
	*Clematis pinnata*	*Clematis heracleifolia*	*Clematis brevicaudata*	–3.81	1.00	0.18
	***Clematis brevicaudata***	***Clematis pinnata***	***Clematis heracleifolia***	**14.12**	**0.00**	**0.56**
SZL	*Clematis pinnata*	*Clematis tubulosa*	*Clematis brevicaudata*	–2.14	0.98	–0.26
	*Clematis pinnata*	*Clematis brevicaudata*	*Clematis tubulosa*	–2.52	0.99	0.13
	***Clematis tubulosa***	***Clematis pinnata***	***Clematis brevicaudata***	**14.20**	**0.00**	**0.54**
WGY	*Clematis pinnata*	*Clematis heracleifolia*	*Clematis brevicaudata*	–12.20	1.00	0.34
	*Clematis pinnata*	*Clematis brevicaudata*	*Clematis heracleifolia*	–5.86	1.00	13.10
	***Clematis heracleifolia***	***Clematis pinnata***	***Clematis brevicaudata***	**11.27**	**0.00**	**0.33**
YMS	*Clematis pinnata*	*Clematis brevicaudata*	*Clematis tubulosa*	–1.43	0.92	0.074
	*Clematis pinnata*	*Clematis tubulosa*	*Clematis brevicaudata*	–1.31	0.91	–0.10
	***Clematis brevicaudata***	***Clematis pinnata***	***Clematis tubulosa***	**16.36**	**0.00**	**0.48**

*?, with unclear identification.*

*, Population names see Table 2.

**, HyDe performs a formal statistical test of γ = 0 versus γ > 0 using Z-test. The higher the Z-score, the more reliable of a hybrid event.

***, According to Meng and Kubatko’s (2009) hybrid model, a hybrid taxa is either sister to “P1” with probability (γ) or sister to “P2” with probability (1–γ), the null hypothesis was that when hybridization was absent, the expected value of γ should be 0.

*Bold rows show possible hybridization events.*

### Chloroplast Genome Analysis

We obtained about 4 Gb of clean NGS data from each sample and filtered 132,798–443,873 cp reads for *C.* × *pinnata* samples and its close relatives by *de novo* assembly. Chloroplast genome sizes of *C.* × *pinnata*, *C. brevicaudata*, *C. heracleifolia*, and *C. tubulosa* ranged from 159,597 bp (*C. tubulosa* from YMS) to 159,667 bp (*C. brevicaudata* from YMS) and the overall GC content of all the four species were around 38%. All the acquired plastome sequences consisted of a pair of IRs (31,038–31,087 bp), separated by an LSC (79,392–79,419 bp) and an SSC (18,093–18,187 bp) regions (Supplementary Figure S6). The cp genomes of all the four *Clematis* species encoded an identical set of 112 genes, including 18 genes with introns, 78 protein-coding, 29 tRNA and four rRNA genes, and 25 genes are in IR region. Structural variation of the newly sampled cp genomes, such as gene inversion/translocation and IR expansion, was similar to that previously reported for other *Clematis* species (Liu et al., 2018).

**FIGURE 5:**
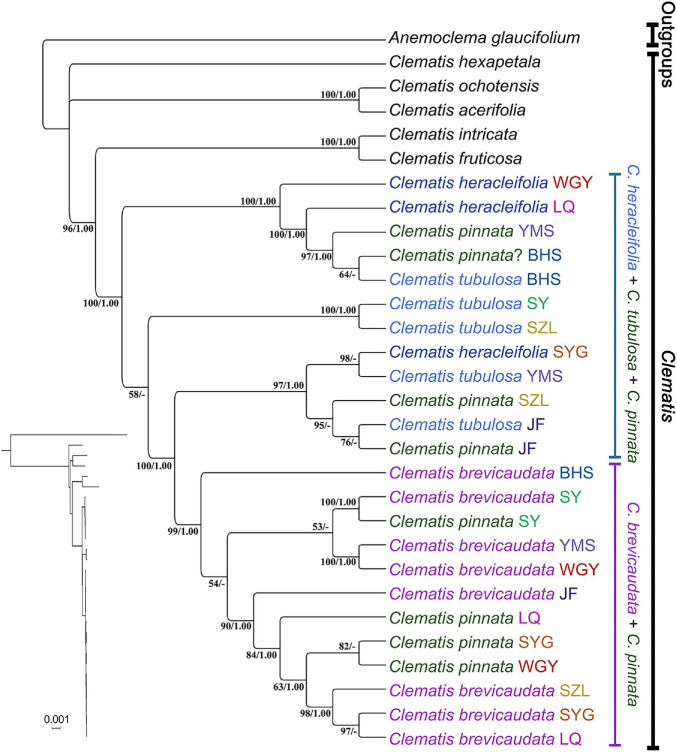
Phylogeny of eight populations (one individual for each population) of *Clematis pinnata* and its close relatives inferred from complete plastome sequences using maximum likelihood and Bayesian inference methods. Bayesian phylogram is shown left below and the dendrogram with ML bootstrap values/posterior probability values are shown at right side. Population location name abbreviations are explained in Table 2. – shows that support values of ML bootstrap values are less than 50 and Bayesian PP values are less than 0.95.

Chloroplast phylogenomic analysis showed that all the samples of *C.* × *pinnata* and its possible progenitors formed a strongly supported clade (ML BS = 100, PP = 1, Figure 5). Within this clade, the branch lengths of subclades and terminal branches are very short, and the resolution as well as support values within the clade are relatively low. Individuals of *C.* × *pinnata* from different sites were separated from each other. Some *C.* × *pinnata* samples grouped with *C. brevicaudata* and clustered into a subclade (ML BS = 99, PP = 1), whereas others grouped with *C. tubulosa* and *C. heracleifolia* and were paraphyletic to the *C. brevicaudata-C.* × *pinnata* subclade. Unlike the nuclear phylogeny, the cp genomic phylogeny showed that both *C. heracleifolia* and *C. tubulosa* are not monophyletic.

### Morphological Analyses

Qualitative characters suggested that *C.* × *pinnata* exhibited uniparental character states (e.g., hairs on both sides of lamina are the same with *C. brevicaudata*, hair of inside sepal is more similar to *C. heracleifolia*/*C. tubulosa*), intermediate character states (e.g., hair on stamens, sepal color, and spreading direction of the sepals), and new character states to its progenitors (e.g., leaf type) (Supplementary Table S3).

**FIGURE 6:**
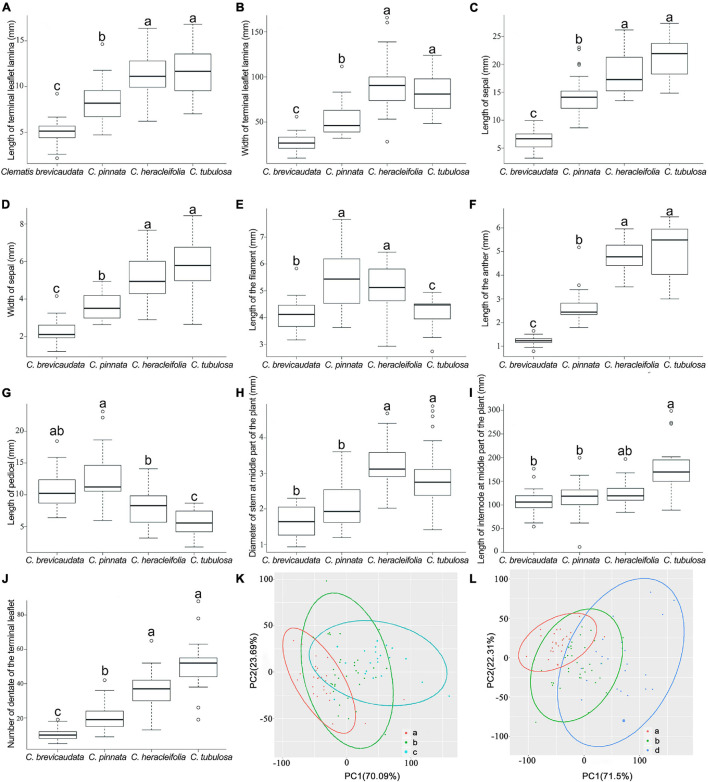
Boxplots **(A–J)** and PCA plots **(K,L)** based on ten quantitative morphological characters of *Clematis pinnata* and its putative parents. **(A–J)** In each boxplot, groups sharing letters denote no significant difference between those groups, as identified by *post hoc* Tukey’s tests with false discovery rate corrections (Benjamini and Hochberg, 1995) for multiple comparisons. **(K,L)** Venn diagrams of PCA results show *C. pinnata*’s (b, 29 specimens) quantitative characters are intermediate to either *C. brevicaudata* (a, 124 specimens) and *C. heracleifolia* (c, 56 specimens) or to *C. brevicaudata* and *C. tubulosa* (d, 69 specimens).

Eight of the ten quantitative characters showed that *C.* × *pinnata* measures fell in between *C. brevicaudata* and *C. heracleifolia*/*C. tubulosa* (Figures 6A–D,F,H–J). Whereas, the other two characters, pedicel and filament lengths, were larger in *C.* × *pinnata* than in its putative progenitors (Figures 6E,G). PCA results showed that *C.* × *pinnata*’s quantitative characters are intermediate to either *C. brevicaudata* and *C. heracleifolia* or to *C. brevicaudata* and *C. tubulosa* (Figures 6K,L).

### Ecological Niche Modeling and Niche Overlapping Test

After removing overly correlated variables (*r* < 0.7) (Guisan and Thuiller, 2005), we reserved nine ecological variables for niche modeling (Supplementary Table S6). The AUC values of the four tested species ranged between 0.986 (*C. brevicaudata*) and 0.998 (*C.* × *pinnata*), indicating that the models had high levels of accuracy and validity. The predicted suitable habitats of the four species are shown in Supplementary Figure S7. Jackknife results (Supplementary Table S10) revealed that UVB4 and BIO18 were important for all the species, whereas BIO8 and BIO19 were only important for *C. brevicaudata*; UVB1 was important for *C.* × *pinnata*, *C. heracleifolia* and *C. tubulosa*; BIO15 was a key variable for *C.* × *pinnata* and *C*. *tubulosa*; and Soil pH was important for *C. heracleifolia*. The top four key environmental variables for *C.* × *pinnata* were the same as those for *C. tubulosa*.

Both niche equivalency test and PCA-env results rejected the null hypothesis that ecological niches for *C. brevicaudata* vs. *C. heracleifolia* and *C. brevicaudata* vs. *C. tubulosa* were overlapping (*p* < 0.05). Whereas, the hypothesis of niches overlapping of *C.* × *pinnata* vs. *C. heracleifolia*, *C.* × *pinnata* vs. *C. tubulosa*, *C.* × *pinnata* vs. *C. brevicaudata*, and *C. heracleifolia* vs. *C. tubulosa*, was accepted by the two statistical analyses (Table 4).

**TABLE 4:** Niche overlap statistics between tested species pairs based on PCA-env and the equivalency test using Schoener’s *D* (Schoener, 1968) and Warren’s *I* (Warren et al., 2008).

	PCA-env: the equivalency test		MaxEnt: the equivalency test	
		
	D	I	D	I
*C. brevicaudata* v. *C. pinnata*	0.04	0.18	0.38	0.66
*C. brevicaudata* v. *C. heracleifolia*	0.12*	0.32*	0.33*	0.53*
*C. brevicaudata* v. *C. tubulosa*	0.09**	0.27**	0.41*	0.70*
*C. pinnata* v. *C. tubulosa*	0.46	0.67	0.43	0.69
*C. pinnata* v. *C. heracleifolia*	0.39	0.61	0.26	0.52
*C. heracleifolia* v. *C. tubulosa*	0.62	0.71	0.29	0.50

*p < 0.05, **p < 0.01.

## Discussion

In this study, we present the first comprehensive study on the natural hybrid origin of *Clematis* species. Testing hybrid hypothesis and species status of *C.* × *pinnata* is the first important step for resolving its taxonomic problems, and hence obtains deeper insights into the diversification of the genus.

### Phylogenomic Analysis Confirms Hybrid Origin of *Clematis* × *pinnata*

The application of genomic data has become an effective means to validate hybridization events. Many natural hybridization events have been proposed and validated in numerous organisms (Roberts and Roalson, 2018; Cao et al., 2019; Glémin et al., 2019; Wang et al., 2019; Zhang et al., 2019; Yang et al., 2020). In this study, our nuclear and cp genome analyses confirmed hybrid origin of *C.* × *pinnata* between *C. brevicaudata* and *C. heracleifolia/C. tubulosa* (Figures 2–5). Hybrid origin of *C.* × *pinnata* was further validated by PhyloNet and HyDe analyses, which have been designed for inferring hybridization (Blischak et al., 2018; Wen et al., 2018). Using populational transcriptome data, we not only confirmed hybrid origin of *C.* × *pinnata* but also obtained its parental genetic contributions in all the sampling sites except BHS (Table 3). From HyDe analysis, the genetic contributions of the progenitors of most populations were nearly equal, thus strongly suggesting that most plants of *C.* × *pinnata* represent the F1 of the parents, because segregation may occur in F2 or later generations causing significant biased parental contributions (Soltis and Soltis, 2009). Meanwhile, for the dubious sample from BHS, PhyloNet analysis failed to detect hybridization of this sample and that it clustered closely with *C. brevicaudata*. This result was further confirmed by HyDe analysis using the reduced SNP data (Table 3). The sample from BHS may represent a morphologically variable individual of *C. brevicaudata*

There are some methodological limitations in this study that should be concerned. The complete SNP data may generate biased results due to linkage disequilibrium (LD). In this study, our strategy (randomly select one SNP from each SCOG) greatly relieved the influence of LD. However, because there are no high-quality whole genome references for *Clematis*, and some of the 3170 SCOGs may be still physically closely related, it is possible that the effects of LD still exist. Furthermore, separating hybridization signal from other sources of incongruence, such as incomplete lineage sorting (ILS) and ancestral polymorphism in population, is difficult (Maddison, 1997). The ABBA-BABA tests implemented in HyDe analysis (or D-statistic test, Patterson et al., 2012) may fail to distinguish signal between hybridization and ancient population structure using SNP data, because this test do not test for hybridization *per se*, but for deviations from the Wright–Fisher model of random mating which can occur as a result from many different evolutionary processes (Huynh et al., 2019).

Another issue is concerned with our RNA-seq sampling. In the eight collecting sites, we collected multiple *C.* × *pinnata* individuals from five sites (Table 2). These multiple individuals from each site were mixed together for RNA-seq to represent genetic features in each population. However, in view that each *C.* × *pinnata* individual may have evolved independently, mixed samples may obtain inaccurate or incorrect result, especially when backcrossing occurred in some individuals. Further analysis using transcriptome data from separate individuals should be conducted to review the impact of this mixed sampling strategy.

### Complete Chloroplast Genome Analysis Indicate Multiple Hybrid Directions

In recent years, complete cp genomes have been widely used in plant phylogenetic reconstructions (e.g., Moore et al., 2010; Li et al., 2019). Because cp genomes show uniparental inheritance in most angiosperm species, it is also a good molecular marker for inferring hybridization and introgression (Liu et al., 2020). In this study, the cp genome analysis demonstrated that multiple *C.* × *pinnata* samples do not form a single lineage (Figures 2, 3, 5). From cp genome phylogeny, we can clearly tell which one acted as the maternal parent of *C.* × *pinnata* (Figure 5). The result showed that all the three putative parental species may have contributed maternally to *C.* × *pinnata*. This results also depicted that different *C.* × *pinnata* individuals evolved from different hybrid events.

### Causes and Consequences of Natural Hybridization Between *Clematis* Species

From our FCM and phylogenomic results, individuals of *C.* × *pinnata* are homoploid hybrids between *C. brevicaudata* and *C. heracleifolia/C. tubulosa*. Different hybridization events happened in different distribution sites, and plants of *C.* × *pinnata* may be predominantly F1 individuals. This conclusion is also supported by several lines of evidence. Our field investigations found that *C.* × *pinnata*’s individuals always occur in the places where both *C. brevicaudata* and *C. heracleifolia/C. tubulosa* are present. The flowering time of all four taxa overlap from July to September, thus creating the potential for cross-pollination. For this reason, if *C.* × *pinnata* is a species, it cannot easily survive from the parental introgression. In this condition, homoploid hybrid speciation is often accompanied by ecological isolation between daughter species and its parental species (Liu et al., 2014; Kadereit, 2015; White et al., 2018). However, the niche modeling and the niche equivalency test results demonstrated that *C.* × *pinnata* has not adapted to a new ecological niche independent of its parents’. From long-term observation, we found that *C.* × *pinnata* suffers from reduced fertility of a high proportion of pollen and fruit abortion, which was also reported by Shi (2003). For example, a *C.* × *pinnata* individual in Jiufeng, Beijing, noted only a few normal achenes per year, while most of them did not develop at all.

Except for several prerequisites for natural hybridization, e.g., close kinship, overlapping distribution, similar flowering period, shared pollinators, and same chromosome numbers (Ning et al., 2019), habitat disturbance has been often considered as one of the most important factors promoting hybridization (Arnold, 1997; Li et al., 2017; Liao et al., 2021). In the study area of Beijing and Liaoning Province, we found all the *C.* × *pinnata* individuals occurred along the mountain roadside. Whereas, *C. brevicaudata* and *C. heracleifolia/C. tubulosa* can occupy much larger distribution ranges than *C.* × *pinnata*. In autumn, the mountain roadside area will be cleared by forest workers for fire prevention in northern China. This provides opportunity for the parental species to contact each other, and open up habitat for the new hybrids (Li et al., 2017).

Recent studies have shown that F1 hybrids are common in angiosperms and that they can successfully impede gene flow and thus maintain species boundaries between parental species especially in areas where habitat disturbance is high (Liao et al., 2021). Morphologically, *C.* sect. *Clematis* and sect. *Tubulosae* have been considered distantly related in the genus due to their great morphological divergence. They were often placed into different subgenera due to their different floral characters (Tamura, 1995; Grey-Wilson, 2000). Only recently have molecular phylogenetic studies clarified their close relationship (Xie et al., 2011; Lehtonen et al., 2016; Yan et al., 2016; He et al., 2021). Representative species of these two sections, *C. brevicaudata* and *C. heracleifolia/C. tubulosa* shared part of their distribution areas in Beijing, Hebei, and Liaoning Provinces, which are exactly the distribution ranges of *C. pinnata*. The presence of *C.* × *pinnata* (F1 hybrid, with reduced fertility) can impede gene flow between parental species, and maintain species boundaries of *C. brevicaudata* and *C. heracleifolia/C. tubulosa* in their contact zones.

In *Clematis*, other species like *C. ochotensis* (sect. *Atragene*) also showed hybrid origin from other two different sections (sect. *Montana* and sect. *Fruticella* and/or sect. *Meclatis*, Figure 4). However, we neither have morphological evidence nor sufficient sampling to discuss about this hybridization event. This study demonstrates that interspecific hybridization between two morphologically highly diverged species can occur naturally in *Clematis*, and hybridization may play an important role in the evolution and diversification of the genus. Taxonomy of *Clematis* may have suffered from widely hybridization among morphologically diverged species.

### Morphology, Species Status, and Taxonomy of *Clematis* × *pinnata*

This study provided opportunity to investigate how morphological characters of hybrids can be expressed in comparison with its morphologically highly diverged parents. Hybrids are often expected to be morphologically intermediate. However, morphological analyses of natural and artificial hybrids showed that characters of hybrids can be truly intermediate, or identical to those of either paternal or maternal parent, or even new traits (Soltis and Soltis, 2009). In this study, our morphological analysis showed that *C.* × *pinnata* exhibited all kinds of morphological outcomes, such as intermediate characters, uniparental characters, and new characters compared to its parental species (Supplementary Table S3 and Figure 6).

*Clematis* × *pinnata* shows extensive variation in morphological characters especially in its leaf types and shapes. Leaves of *C.* × *pinnata* are predominantly pinnate with five leaflets, but ternate, bi-ternate, or even simple leaves sometimes also occur in different development stages of the plants (Xie et al., 2005). Morphological characters often exhibit higher variability in hybrids than in hybrid species (Zhang et al., 2020). High morphological variation in *C.* × *pinnata* also supports the point that *C.* × *pinnata* represent early generation of hybrids rather than a hybrid species.

Our morphological analysis did not clearly distinguish *C. heracleifolia* from *C. tubulosa*. Except some qualitative characters, e.g., pedicel length, sepal shape, and pollen type (Wang and Xie, 2007), other morphological characters failed to distinguish the two species from one another. Furthermore, our genome analysis and niche equivalency test also did not clearly separate them. These results raise an interesting issue of species delimitation in *C.* sect. *Tubulosae* that need to be studied in the future.

All the previous taxonomic studies recognized *C.* × *pinnata* as a distinct species based solely on morphology (Fang, 1980; Xie et al., 2005; Wang and Xie, 2007). However, our study has clearly shown that plants of *C.* × *pinnata* has not formed a self-evolving lineage, and are generated by recurrent hybridization events between *C. brevicaudata* and *C. heracleifolia/C. tubulosa* in their overlapping zones. *Clematis* × *pinnata* cannot hold species status from our analysis, and we make a taxonomic treatment as below.

## Taxonomic Treatment

***Clematis* × *pinnata*** Maxim. (pro sp.) (= *Clematis brevicaudata* DC.: ♀or ♂ × *C. heracleifolia* DC.: ♀or ♂, *C. brevicaudata* DC.: ♀or ♂ × *C. tubulosa* Turcz.: ♀or ♂) in Bull. Acad. Imp. Sci. Saint-Pétersbourg, sér. 3. 22: 216. 1876 – Holotype: China. Near Beijing, ca. 1845, *A.A. Tatarinov s. n.* (LE!; isotype: PE!)

= *C. tatarinowii* Maxim., in l.c. Holotype: China. Beijing, ca. 1845, *A.A. Tatarinov s. n.* (LE; PE [photo!]; isotype: PE!)

= *C. pinnata* Maxim. var. *tatarinowii* (Maxim.) Kuntze in Verh. Bot. Vereins Prov. Brandenburg 26(2): 182. 1885.

= *C. pinnata* Maxim. var. *ternatifolia* W.T. Wang in Acta Phytotax. Sin. 39(4): 331. 2001. Holotype: China. Beijing, Pinggu, Mt. Nanjishan, 13. June. 1972, *Pinggu Exped. 224.* (PE!)

*Distribution*. – China. Beijing and adjacent areas of Hebei Province and Tianjin, and central Liaoning Province. Wang and Xie (2007) recorded that *C.* × *pinnata* occurred in Heilongjiang Province based on a single collection of *E. Licent 9221* (collected in 20 August 1929) deposited in Tianjin Natural History Museum (TIE). However, we carefully checked the specimen in TIE and found that this specimen was collected in Yangjiaping, Zhuolu County, Hebei Province near the western border of Beijing. So, we don’t have evidence that *C.* × *pinnata* is distributed in Heilongjiang Province.

## Data Availability Statement

The datasets presented in this study can be found in online repositories. The name of the repository and accession number can be found below: National Center for Biotechnology Information (NCBI) BioProject, https://www.ncbi.nlm.nih.gov/bioproject/, PRJNA657443.

## Author Contributions

RL, JH, YL, and LlL analyzed the data and prepared the draft. MY, RL, YL, SY, JC, LqL, and LX conducted the field surveys. JC, LP, LqL, and LX proposed the hybrid hypothesis and designed the study. JC and LX wrote and revised the manuscript. All the authors contributed to the article and approved the submitted version.

## Conflict of Interest

LP was employed by company Beijing Forestry University Forest Science Co. Ltd. The remaining authors declare that the research was conducted in the absence of any commercial or financial relationships that could be construed as a potential conflict of interest.

## Publisher’s Note

All claims expressed in this article are solely those of the authors and do not necessarily represent those of their affiliated organizations, or those of the publisher, the editors and the reviewers. Any product that may be evaluated in this article, or claim that may be made by its manufacturer, is not guaranteed or endorsed by the publisher.

## Footnotes

1
http://www.biomarker.com.cn/

2
https://github.com/HeJian151004/get_homology

3
https://cvalues.science.kew.org/

4
https://github.com/Jhe1004/SNPRandomEextraction

5
http://hannonlab.cshl.edu/fastx_toolkit

6
https://github.com/HeJian151004/get_homology

7
http://www.cvh.ac.cn

8
https://www.google.com/earth

9
http://www.worldclim.org

10
https://www.ufz.de/gluv/index.php?en=32435

11
http://www.fao.org/soils-portal/soil-survey/soil-maps-and-databases/harmonized-world-soil-database-v12/zh/

12
http://nelson.wisc.edu/sage/data-and-models/atlas/maps.php

13
https://www.gadm.org/maps.html

14
http://biodiversityinformatics.amnh.org/open_source/maxent/

15
https://www.r-project.org/

## Supplementary Material

Supplementary Figure S1 Natural distribution (blue dash circles) and collecting sites (points) of *Clematis pinnata*.

Supplementary Figure S2 Peak value image based on flow cytometry analysis of *Clematis pinnata*, *C. tubulosa*, and *C. heracleifolia* using *C. brevicaudata* as the external reference.

Supplementary Figure S3 The backbone of *Clematis* phylogeny inferred from the concatenated and coalescent based method using 3170 co-orthologous genes with exclusion the two putative taxa, *C. pinnata* and *C. ochotensis*. ML bootstrap values (MLBS) of the concatenated analysis were marked on the branches with ^∗^ indicating MLBS = 100.

Supplementary Figure S4 Optimal species networks of the eight populations of *Clematis pinnata* and its putative parents, as well as of closely related species, inferred using PhyloNet. The results display maximum pseudolikelihood trees with maximum zero to four reticulations allowed. Bar charts show probabilities of different maximum numbers of reticulations. Population location name abbreviations are explained in Table 2.

Supplementary Figure S5 Optimal species networks of the BHS populations of *Clematis pinnata* and its putative parents, as well as of closely related species, inferred using PhyloNet with exclusion of another putative hybrid species *C. ochotensis*. The results display maximum pseudolikelihood trees with maximum zero to four reticulations allowed. Bar charts show probabilities of different maximum numbers of reticulations. Population location name abbreviations are explained in Table 2.

Supplementary Figure S6 Chloroplast genome maps for *Clematis pinnata*. Thick lines on the complete outer circle identify the inverted repeat regions (IRa and IRb). The innermost track of the plastome shows the G + C content. Genes on the outside of the map are transcribed in a clockwise direction and genes on the inside of the map are transcribed in a counter-clockwise direction. INV, inversion; TP, transposition; IR, inverted repeats; LSC, large single copy; SSC, small single copy; Pi, nucleotide variability.

Supplementary Figure S7 Potential suitable distribution areas for *Clematis pinnata* and its putative parent species as predicted using MaxEnt modeling.

Supplementary Table S1 Information of transcriptome data of *Clematis pinnata* and its relatives sampled in this study.

Supplementary Table S2 Sampling information for the newly generated genome skimming data.

Supplementary Table S3 Measurements and statistical summaries of 30 selected morphological characters of *Clematis pinnata* and its putative parents.

Supplementary Table S4 Samples of *Clematis pinnata* and its putative parents for pollen morphology scanning using Scanning Electron Microscopy (SEM).

Supplementary Table S5 Specimens’ information of *Clematis brevicaudata*, *C. heracleifolia*, *C. tubulosa*, *C. pinnata* used in morphological and niche modeling analysis.

Supplementary Table S6 The 33 environmental variables used for the niche modeling.

Supplementary Table S7 Flow cytometry analysis of *Clematis tubulosa* and *C. pinnata* using *C. brevicaudata* as an external standard reference.

Supplementary Table S8 Flow cytometry analysis of *Clematis heracleifolia* using *C. brevicaudata* as an external standard reference.

Supplementary Table S9 HyDe analysis of *Clematis pinnata* and its putative parents in each population using the complete SNP data. Sample size of *C. pinnata*: eight populations, 28 individuals.

Supplementary Table S10 Percentage contributions and permutation importance of environmental variables included in MaxEnt modeling for *Clematis pinnata* and its putative parents.
