# Supplementary figures and images for: Natural Hybrid Origin of the Controversial “Species” Clematis × pinnata (Ranunculaceae) Based on Multidisciplinary Evidence
Source: Front Plant Sci. 2021 Oct 12;12:745988. doi: 10.3389/fpls.2021.745988 (PMC8545901; doi:10.3389/fpls.2021.745988)

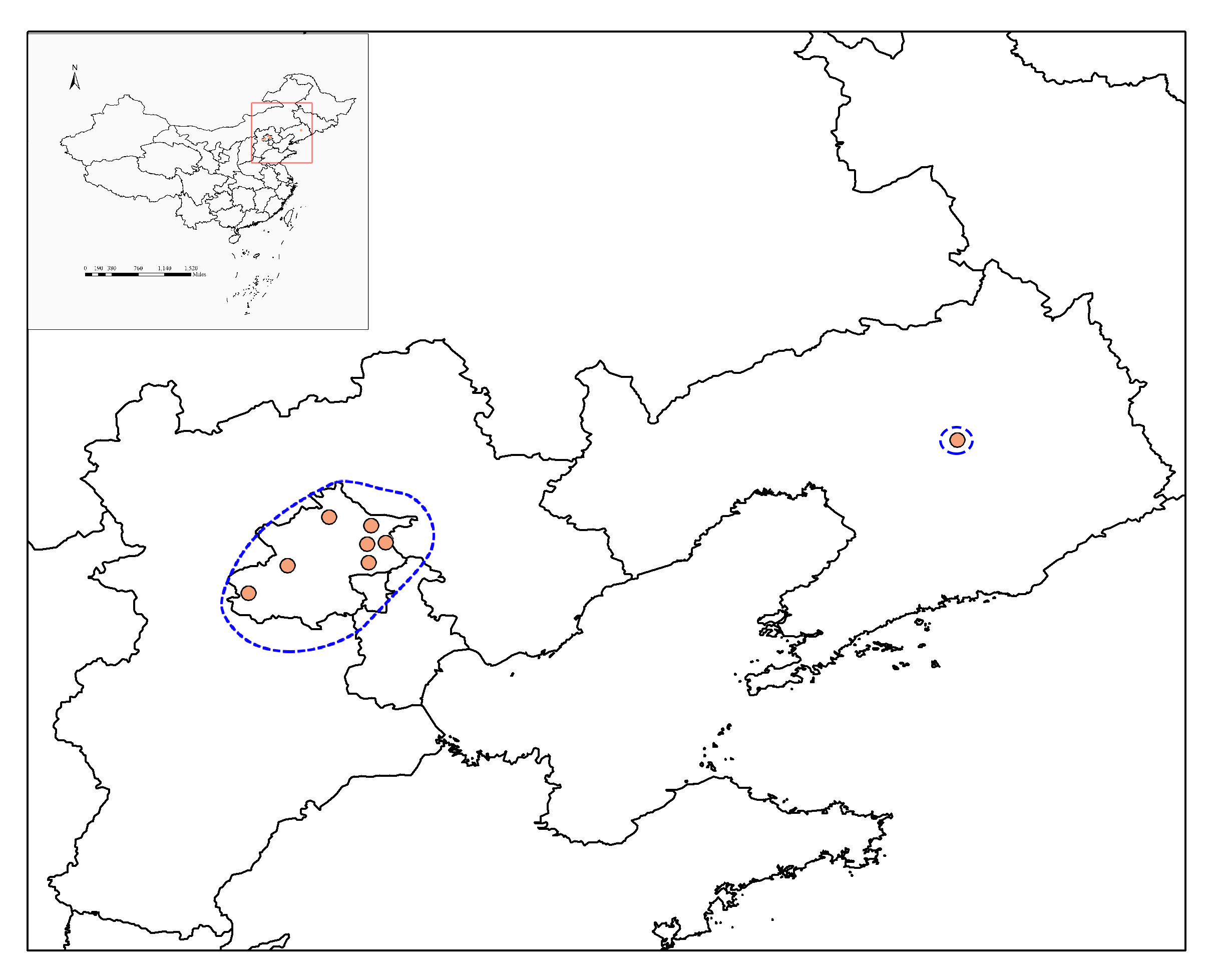

Supplement: Supplementary Figure S1 — Natural distribution (blue dash circles) and collecting sites (points) of Clematis pinnata. [file Image_1.TIF]

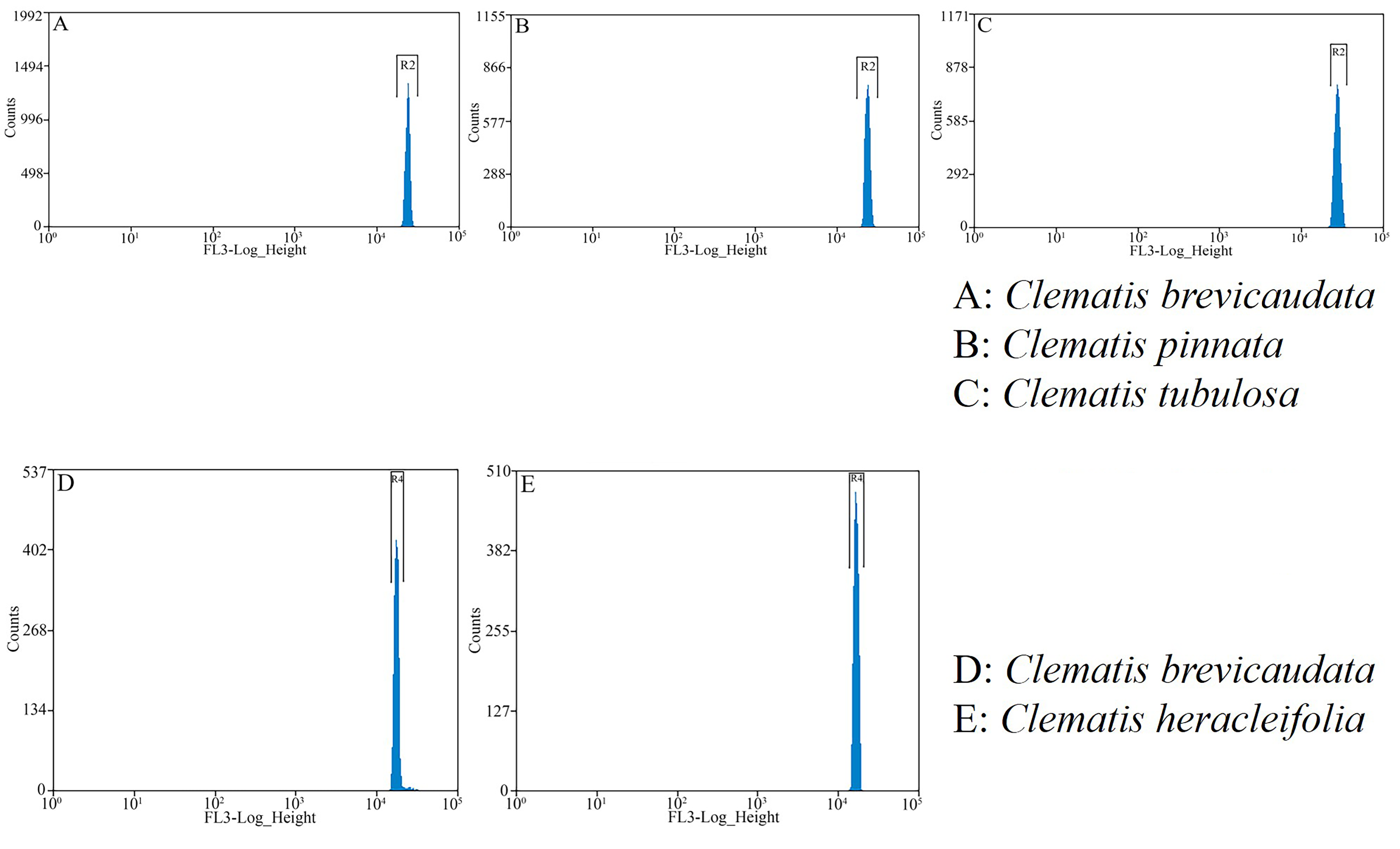

Supplement: Supplementary Figure S2 — Peak value image based on flow cytometry analysis of Clematis pinnata, C. tubulosa, and C. heracleifolia using C. brevicaudata as the external reference. [file Image_2.TIF]

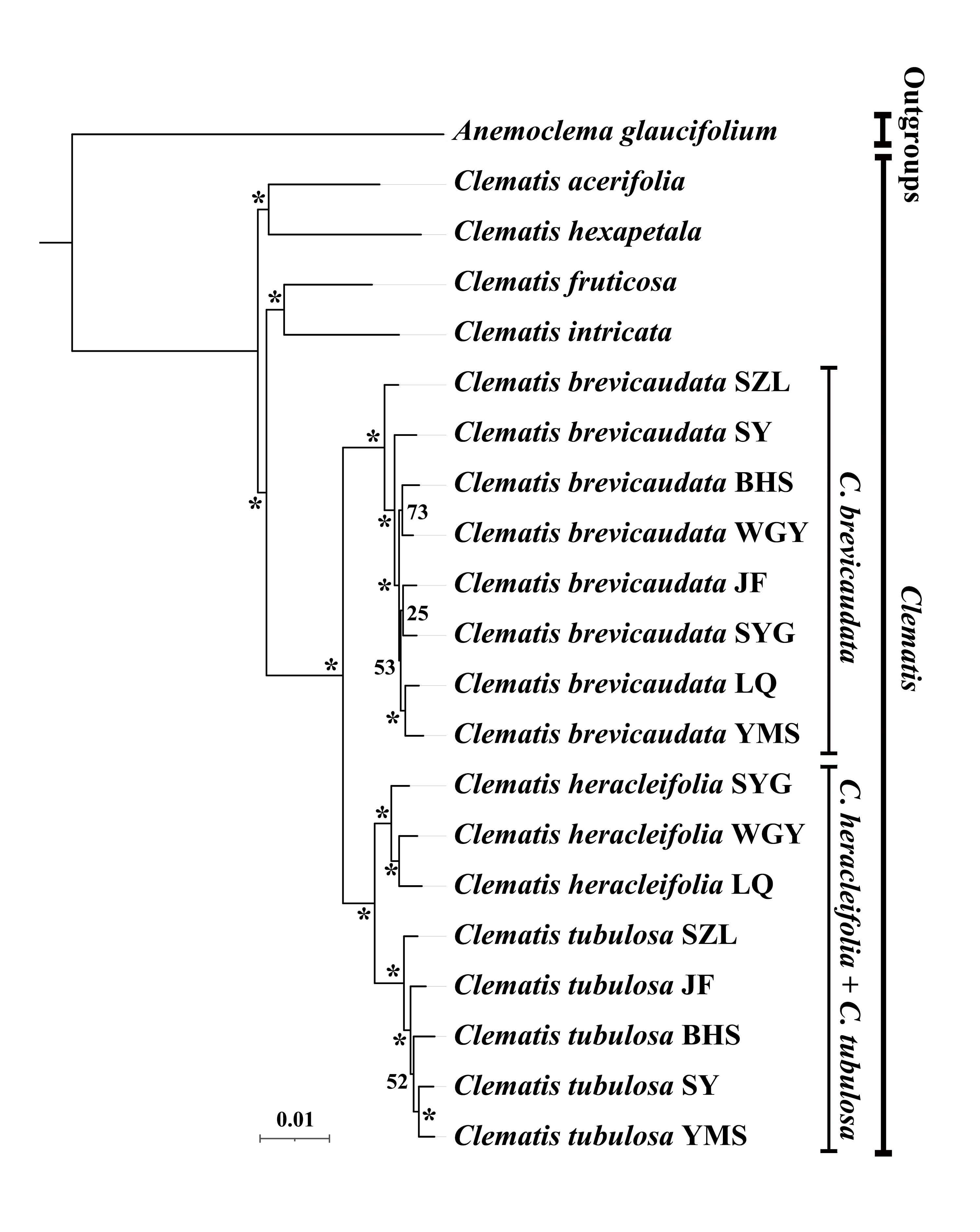

Supplement: Supplementary Figure S3 — The backbone of Clematis phylogeny inferred from the concatenated and coalescent based method using 3170 co-orthologous genes with exclusion the two putative taxa, C. pinnata and C. ochotensis. ML bootstrap values (MLBS) of the concatenated analysis were marked on the branches with ∗ indicating MLBS = 100. [file Image_3.JPEG]

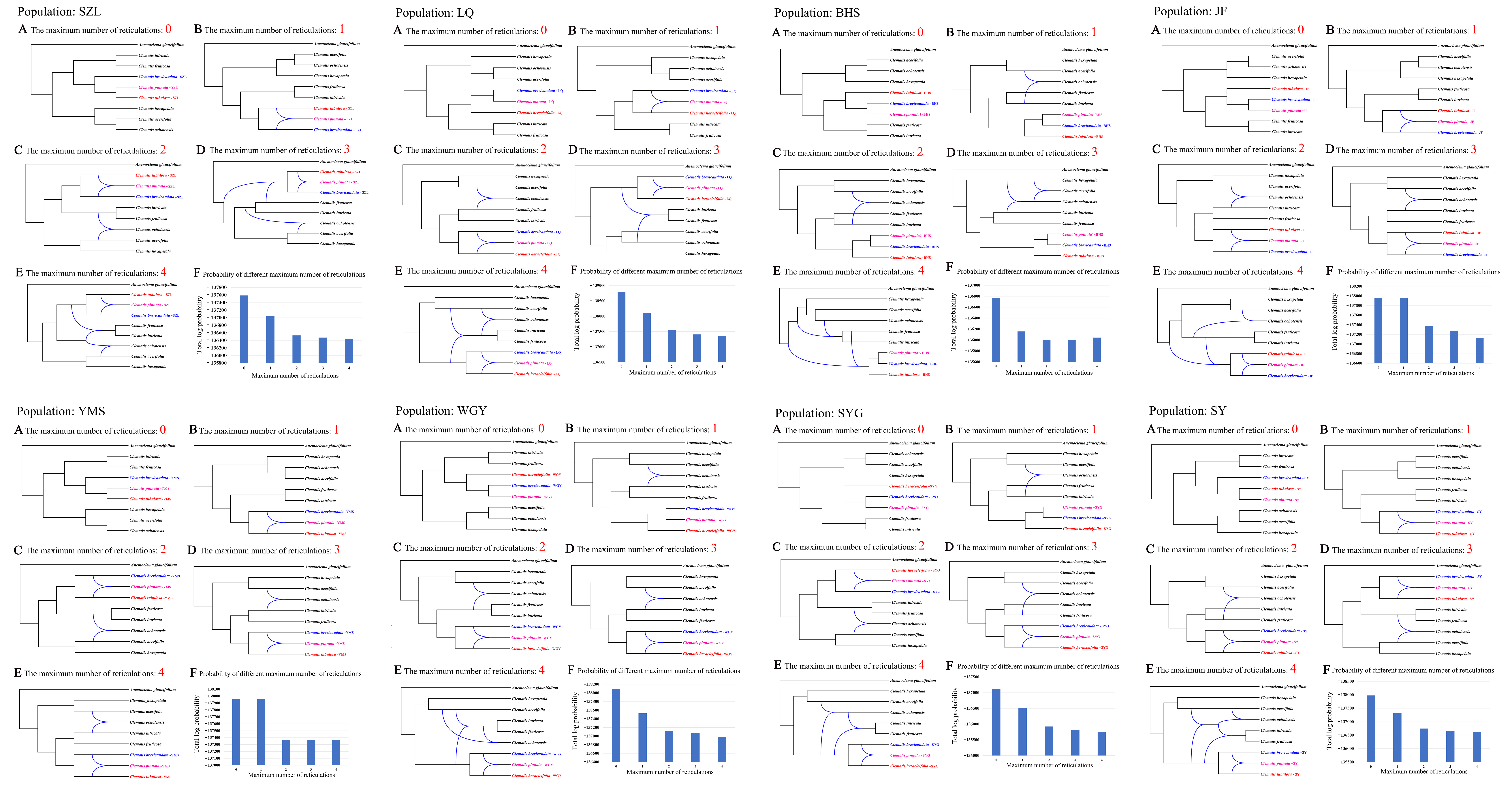

Supplement: Supplementary Figure S4 — Optimal species networks of the eight populations of Clematis pinnata and its putative parents, as well as of closely related species, inferred using PhyloNet. The results display maximum pseudolikelihood trees with maximum zero to four reticulations allowed. Bar charts show probabilities of different maximum numbers of reticulations. Population location name abbreviations are explained in Table 2. [file Image_4.JPEG]

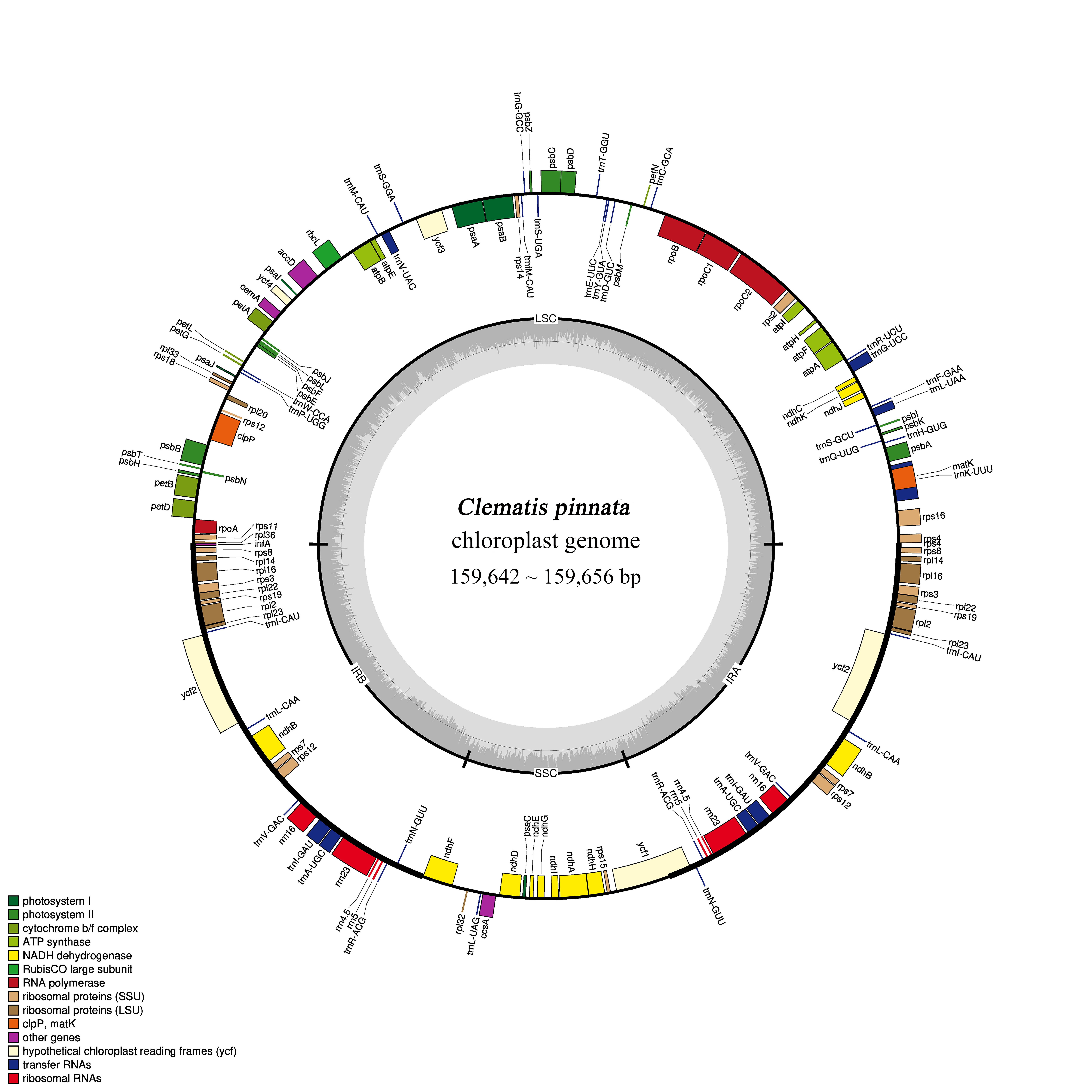

Supplement: Supplementary Figure S6 — Chloroplast genome maps for Clematis pinnata. Thick lines on the complete outer circle identify the inverted repeat regions (IRa and IRb). The innermost track of the plastome shows the G + C content. Genes on the outside of the map are transcribed in a clockwise direction and genes on the inside of the map are transcribed in a counter-clockwise direction. INV, inversion; TP, transposition; IR, inverted repeats; LSC, large single copy; SSC, small single copy; Pi, nucleotide variability. [file Image_6.TIF]

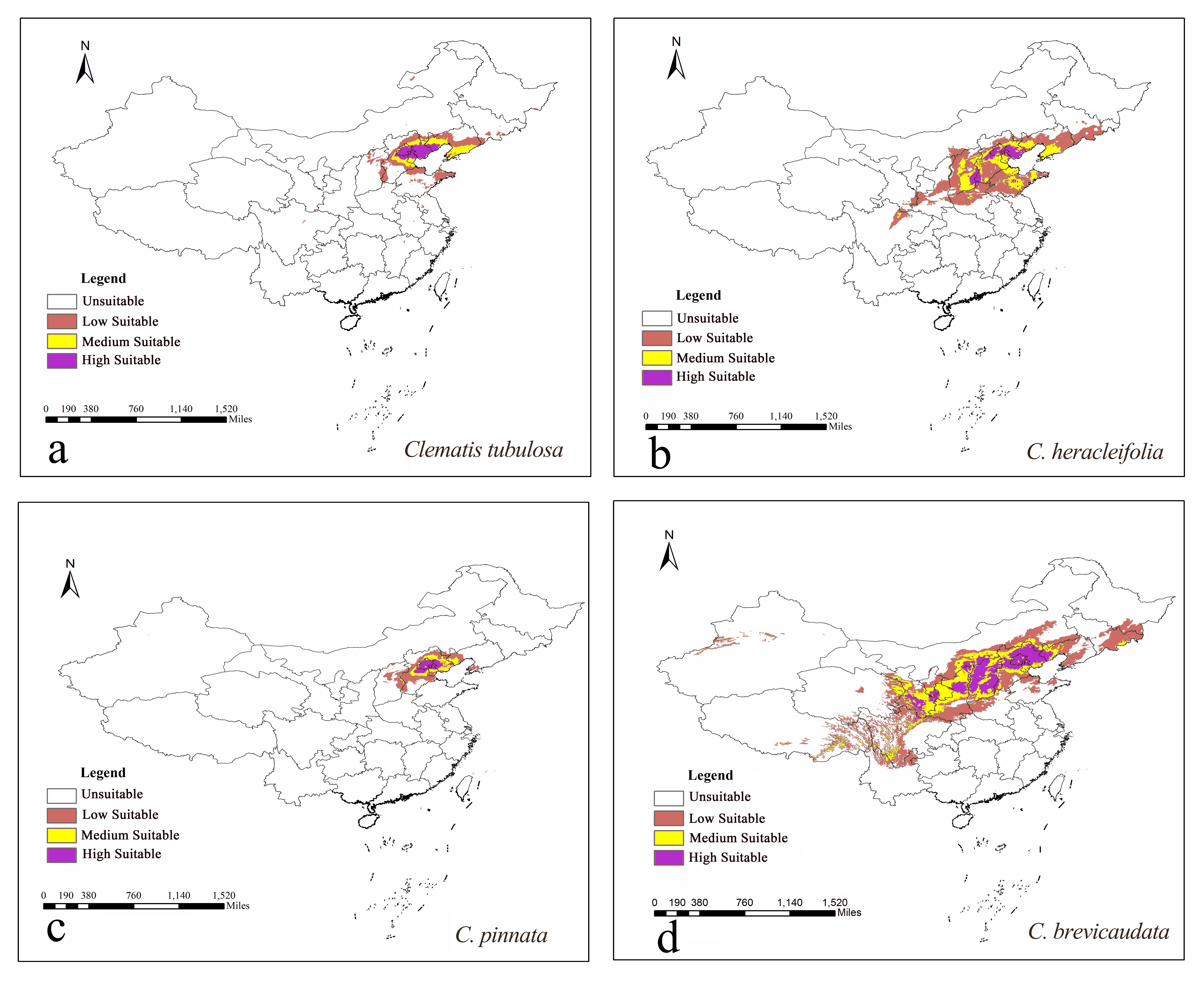

Supplement: Supplementary Figure S7 — Potential suitable distribution areas for Clematis pinnata and its putative parent species as predicted using MaxEnt modeling. [file Image_7.JPEG]
