# Supplementary material for: Natural Hybrid Origin of the Controversial “Species” Clematis × pinnata (Ranunculaceae) Based on Multidisciplinary Evidence
Source: Front Plant Sci. 2021 Oct 12;12:745988. doi: 10.3389/fpls.2021.745988 (PMC8545901; doi:10.3389/fpls.2021.745988)
Supplement: Supplementary Table S1 — Information of transcriptome data of Clematis pinnata and its relatives sampled in this study. [file Table_1.DOCX]

**TABLE S1.** Information of transcriptome data of *Clematis pinnata* and its relatives sampled in this study.

| Population | Species | Collection number | Number of raw reads | Size of data (GB) | Number of contigs |
| --- | --- | --- | --- | --- | --- |
| Jianchuan, Yunnan, China | *Anemoclema glaucifolium* | L. Xie 20190715-04 | 43, 480, 290 | 7.45 * 2 | 22, 078 |
| Jiufeng, Beijing, China | *Clematis hexapetala* | 1. Xie JF-6 | 43, 633, 066 | 7.47 * 2 | 37, 289 |
| Nanshiyang, Beijing, China | *Clematis acerifolia* | YM004 | 40, 004, 296 | 6.85 * 2 | 20, 001 |
| Zhangjiakou, Hebei, China | *Clematis ochotensis* | L. Xie 2019051801 | 48, 954, 924 | 8.39 * 2 | 18, 352 |
| Baojiakou, Hebei, China | *Clematis intricata* | L. Xie 2019052001 | 42, 635, 936 | 7.31 * 2 | 20, 757 |
| Datong, Shanxi, China | *Clematis fruticosa* | 1. He 20170064 | 46, 251, 607 | 7.92 * 2 | 25, 474 |
| Sizuolou, Beijing, China | *Clematis brevicaudata*-SZL | LRD0133 | 43, 050, 222 | 7.38 * 2 | 20, 197 |
|  | *Clematis tubulosa*-SZL | LRD0058 | 45, 021, 768 | 7.71 * 2 | 24, 429 |
|  | *Clematis pinnata*-SZL | LRD0053 | 43, 388, 852 | 7.43 * 2 | 22, 757 |
| Laoquan, Beijing, China | *Clematis brevicaudata*-LQ | LRD0024 | 42, 656, 430 | 7.30 * 2 | 26, 975 |
|  | *Clematis heracleifolia*-LQ | LRD0009 | 41, 327, 306 | 7.08 * 2 | 20, 171 |
|  | *Clematis pinnata*-LQ | LRD0026 | 43, 043, 038 | 7.38 * 2 | 22, 042 |
| Baihuashan, Beijing, China | *Clematis brevicaudata*-BHS | L.Xie 20190821-01 | 45, 560, 872 | 7.81 * 2 | 21, 006 |
|  | *Clematis tubulosa*-BHS | L.Xie 20190821-02 | 42, 816, 428 | 7.34 * 2 | 21, 649 |
|  | *Clematis pinnata*?-BHS | L.Xie 20190821-03 | 43, 864, 866 | 7.52 * 2 | 29, 080 |
| Jiufeng, Beijing, China | *Clematis brevicaudata*-JF | L.Xie 20180511-01 | 43, 668, 944 | 7.48 * 2 | 20, 445 |
|  | *Clematis tubulosa*-JF | LRD0002 | 43, 761, 746 | 7.50 * 2 | 20, 200 |
|  | *Clematis pinnata*-JF | LRD0008 | 42, 909, 512 | 7.35 * 2 | 21, 969 |
| Yunmengshan, Beijing, China | *Clematis brevicaudata*-YMS | LRD0084 | 42, 897, 606 | 7.35 * 2 | 19, 548 |
|  | *Clematis tubulosa*-YMS | LRD0085 | 43, 121, 824 | 7.39 * 2 | 72, 856 |
|  | *Clematis pinnata*-YMS | LRD0083 | 43, 235, 914 | 7.41 * 2 | 22, 190 |
| Woguayu, Beijing, China | *Clematis brevicaudata*-WGY | LRD0132 | 43, 619, 234 | 7.47 * 2 | 19, 816 |
|  | *Clematis heracleifolia*-WGY | LRD0070 | 45, 473, 044 | 7.79 * 2 | 19, 503 |
|  | *Clematis pinnata*-WGY | LRD0068 | 43, 225, 486 | 7.41 * 2 | 19, 908 |
| Sanyanggu, Beijing, China | *Clematis brevicaudat*a-SYG | LRD0131 | 57, 895, 924 | 9.92 * 2 | 20, 783 |
|  | *Clematis heracleifolia*-SYG | LRD0039 | 44, 145, 392 | 7.56 * 2 | 20, 368 |
|  | *Clematis pinnata*-SYG | LRD0033 | 40, 076, 286 | 6.87 * 2 | 19, 970 |
| Shenyang, Liaoning, China | *Clematis brevicaudata*-SY | LRD0110 | 42, 217, 864 | 7.23 * 2 | 20, 297 |
|  | *Clematis tubulosa*-SY | LRD0106 | 42, 898, 696 | 7.35 * 2 | 34, 275 |
|  | *Clematis pinnata­*-SY | LRD0105 | 41, 999, 124 | 7.20 * 2 | 21, 574 |
