# Supplementary material for: Natural Hybrid Origin of the Controversial “Species” Clematis × pinnata (Ranunculaceae) Based on Multidisciplinary Evidence
Source: Front Plant Sci. 2021 Oct 12;12:745988. doi: 10.3389/fpls.2021.745988 (PMC8545901; doi:10.3389/fpls.2021.745988)
Supplement: Supplementary Table S2 — Sampling information for the newly generated genome skimming data. [file Table_2.DOC]

**TABLE S2.** Sampling information for the newly generated genome skimming data.

| Population | Species | Collection number | Accession No. |
| --- | --- | --- | --- |
| Jianchuan, Yunnan, China | *Anemoclema glaucifolium* | L. Xie 20190715-04 | MH205609 |
| Jiufeng, Beijing, China | *C. hexapetala* | JF-6 | MT876495 |
| Nanshiyang, Beijing, China | *C. acerifolia* | YM004 | NC_039844 |
| Zhangjiakou, Hebei, China | *C. ochotensis* | L. Xie 2019051801 | MT876505 |
| Baojiakou, Hebei, China | *C. intricata* | L. Xie 2019052001 | MT876497 |
| Datong, Shanxi, China | *C. fruticosa* | J. He 20170064 | MT976492 |
| Sizuolou, Beijing, China (SZL) | *C. brevicaudata* | LRD0133 | MT796614 |
|  | *C. tubulosa* | LRD0058 | MT796616 |
|  | *C. pinnata* | LRD0053 | MT796615 |
| Laoquan, Beijing, China (LQ) | *C. brevicaudata* | LRD0024 | MT796605 |
|  | *C. heracleifolia* | LRD0009 | MT796606 |
|  | *C. pinnata* | LRD0026 | MT796607 |
| Baihuashan, Beijing, China (BHS) | *C. brevicaudata* | 20190821-01 | MT796599 |
|  | *C. tubulosa* | 20190821-02 | MT796601 |
|  | *C. pinnata* | 20190821-03 | MT796600 |
| Jiufeng, Beijing, China (JF) | *C. brevicaudata* | 20180511-01 | MT796602 |
|  | *C. tubulosa* | LRD0002 | MT796604 |
|  | *C. pinnata* | LRD0008 | MT796603 |
| Yunmengshan, Beijing, China (YMS) | *C. brevicaudata* | LRD0084 | MT796620 |
|  | *C. tubulosa* | LRD0085 | MT796622 |
|  | *C. pinnata* | LRD0083 | MT796621 |
| Woguayu, Beijing, China (WGY) | *C. brevicaudata* | LRD0132 | MT796617 |
|  | *C. heracleifolia* | LRD0070 | MT796618 |
|  | *C. pinnata* | LRD0068 | MT796619 |
| Sanyanggu, Beijing, China (SYG) | *C. brevicaudata* | LRD0131 | MT796611 |
|  | *C. heracleifolia* | LRD0039 | MT796612 |
|  | *C. pinnata* | LRD0033 | MT796613 |
| Dongling Liaoning, China (SY) | *C. brevicaudata* | LRD0110 | MT796608 |
|  | *C. tubulosa* | LRD0106 | MT796610 |
|  | *C. pinnata* | LRD0105 | MT796609 |
