# Supplementary material for: Natural Hybrid Origin of the Controversial “Species” Clematis × pinnata (Ranunculaceae) Based on Multidisciplinary Evidence
Source: Front Plant Sci. 2021 Oct 12;12:745988. doi: 10.3389/fpls.2021.745988 (PMC8545901; doi:10.3389/fpls.2021.745988)
Supplement: Supplementary Table S3 — Measurements and statistical summaries of 30 selected morphological characters of Clematis pinnata and its putative parents. [file Table_3.DOCX]

**TABLE S3.** Measurements and statistic summaries of the 30 selected morphological characters of *Clematis pinnata* and its putative parents.

| Character | *C. brevicaudata* | *C. pinnata* | *C. heracleifolia* | *C. tubulosa* |
| --- | --- | --- | --- | --- |
| Leaf type | Biternate, once or twice pinnate | 5-foliolately pinnate, sometimes 1-2-ternate or 2-pinnate | ternate | ternate |
| Hair of adaxial leaflet lamina | appressed-puberulous or nearly glabrous | appressed-puberulous or nearly glabrous | subglabrous | subglabrous |
| Hair of abaxial leaflet lamina | appressed-puberulous or nearly glabrous | appressed-puberulous or nearly glabrous | puberulous on veins | puberulous on veins |
| Number of lobes of the terminal leaflet | undivided or 3-lobed | 2-3-lobed or undivided | 3-lobed, rarely undivided | 3-lobed or undivided |
| Hair of stem | puberulous or subglabrous | appressed-puberulous, often glabrescent | appressed-puberulous | densely puberulous |
| Hair of pedicel | puberulous | densely puberulous | densely puberulous | densely velutinous |
| Spreading direction of the sepal | spreading | erect，later spreading | erect | erect |
| Hair of inside sepal | sparsely puberulous | glabrous | glabrous | glabrous |
| Hair of outside sepal | puberulous, velutinous on margin | densely puberulous, velutinous on margin | densely appressed-puberulous, velutinous on margin | appressed-puberulous, velutinous on margin |
| The color of sepal | yellowish-white or white | Whitish purple | blueish-purple | blueish-purple |
| Hair of stamen | puberulous or subglabrous | appressed-puberulous, often glabrescent | appressed-puberulous | densely puberulous |
| Pollen type | tricolpate | tricolpate | tricolpate | pantoporate |
| Hair of filament | glabrous | nearly glabrous | sparsely puberulous or glabrous near apex | sparsely puberulous or glabrous near apex |
| Hair of anther | glabrous | glabrous or pilose | pilose | pilose |
| Length of terminal leaflet lamina (mm) | 51.03±3.44 | 77.54±2.38 | 111.44±4.61 | 121.41±4.61 |
| Width of terminal leaflet lamina (mm) | 27.17±2.24 | 45.03±1.69 | 91.46±5.04 | 86.19±3.07 |
| Ratio of length/width of terminal leaflet lamina | 1.88 | 1.72 | 1.22 | 1.41 |
| Number of dentate of the terminal leaflet | 10.07±0.82 | 16.24±0.70 | 39.13±2.31 | 50.68±1.93 |
| Diameter of stem at middle part of the plant (mm) | 1.81±0.12 | 2.47±0.25 | 3.19±0.13 | 3.17±0.15 |
| Length of internode at middle part of the plant (mm) | 112.37±7.34 | 119.48±5.71 | 129.24±7.16 | 164.54±10.85 |
| Length of pedicel (mm) | 9.77±0.39 | 13.03±0.62 | 9.26±0.74 | 6.52±0.37 |
| Length of sepal (mm) | 7.45±0.21 | 13.98±0.55 | 19.26±0.68 | 20.88±0.53 |
| Width of sepal (mm) | 2.46±0.12 | 3.58±0.14 | 5.36±0.24 | 6.29±0.29 |
| Ratio of length/width of sepal | 3.03 | 3.91 | 3.59 | 3.32 |
| Length of the filament (mm) | 4.54±0.08 | 5.39±0.16 | 5.04±0.14 | 4.1±0.08 |
| Length of the anther (mm) | 1.23±0.03 | 2.51±0.06 | 4.76±0.11 | 4.82±0.14 |
| Ration of length: filament/anther | 3.69 | 2.15 | 1.06 | 0.85 |
| *Polar length (μm) | 23.86±0.13 | abortive | 22.8±0.24 | 21.76±0.17 |
| Equatorial length (μm) | 16.56±0.14 |  | 18.44±0.23 | 21.21±0.18 |
| Ratio of Polar/Equatorial | 1.44 |  | 1.24 | 1.03 |

* We used Scanning Electron Microscopy (SEM) to obtain the detailed pollen characters from *C. pinnata* and its putative parents. Pollen grains came from herbarium specimens. The pollen sac is crushed and adheres directly to the sample table. We used the Hitachi E-1010 ion sputtering instrument to spray gold, observed and photographed under the Hitachi S-3400 scanning electron microscope.
