# Supplementary material for: Natural Hybrid Origin of the Controversial “Species” Clematis × pinnata (Ranunculaceae) Based on Multidisciplinary Evidence
Source: Front Plant Sci. 2021 Oct 12;12:745988. doi: 10.3389/fpls.2021.745988 (PMC8545901; doi:10.3389/fpls.2021.745988)
Supplement: Supplementary Table S4 — Samples of Clematis pinnata and its putative parents for pollen morphology scanning using Scanning Electron Microscopy (SEM). [file Table_4.DOC]

**TABLE S4.** Samples of *Clematis pinnata* and its putative parents for pollen morphology scanning using Scanning Electron Microscopy (SEM).

| Species | Collection number | Locality |
| --- | --- | --- |
| *C. brevicaudata* | JF-07 | Jiufeng forest park, Haidian district, Beijing, China |
| *C. brevicaudata* | LRD0084 | Yunmengshan, Huairou district, Beijing, China |
| *C. heracleifolia* | H17 | Beijing Botanical Garden, Haidian district, Beijing, China |
| *C. heracleifolia* | LRD0015 | Laoquan, Pinggu district, Beijing, China |
| *C. tubulosa* | H9 | Jiufeng forest park, Haidian district, Beijing, China |
| *C. tubulosa* | LRD0087 | Yunmengshan, Huairou district, Beijing, China |
| *C. pinnata* | LRD0053 | Sizuolou, Pinggu district, Beijing, China |
