# Supplementary material for: Natural Hybrid Origin of the Controversial “Species” Clematis × pinnata (Ranunculaceae) Based on Multidisciplinary Evidence
Source: Front Plant Sci. 2021 Oct 12;12:745988. doi: 10.3389/fpls.2021.745988 (PMC8545901; doi:10.3389/fpls.2021.745988)
Supplement: Supplementary Table S5 — Specimens’ information of Clematis brevicaudata, C. heracleifolia, C. tubulosa, C. pinnata used in morphological and niche modeling analysis. [file Table_5.DOCX]

**TABLE S5.** Specimens’ information of *Clematis brevicaudata*, *C. heracleifolia*, *C. tubulosa*, *C. pinnata* used in morphological and niche modelling analysis.

| Taxon | Herbarium | Collection number | Locality | Elevation/m | [Longitude/°](javascript:;) | [Latitude/°](javascript:;) |
| --- | --- | --- | --- | --- | --- | --- |
| *C. brevicaudata* | GH | Makarov s.n. | Primorskiy, Rossiya | 560 | 134.977831 | 44.997586 |
| *C. brevicaudata* | G/GH/MO | Ulanova 5828 | Vladivostok, Primorsky Krai, i-Russia | 160 | 131.938271 | 43.142377 |
| *C. brevicaudata* | GH | E. H. Wilson 8922 | Rankyo, Mozan,Korea | 240 | 125.845054 | 39.082195 |
| *C. brevicaudata* | BNU | Q. R. Liu s.n. | Baihua mountain, mentougou district, Beijing, China | 750 | 115.624647 | 39.850231 |
| *C. brevicaudata* | BJFC | LRD 20180702-11 | Baihua mountain, mentougou district, Beijing, China | 1155 | 115.580721 | 39.840012 |
| *C. brevicaudata* | BJFC | LRD 20180702-12 | Baihua mountain, mentougou district, Beijing, China | 1188 | 115.580935 | 39.835761 |
| *C. brevicaudata* | BJFC | LRD 20180702-13 | Baihua mountain, mentougou district, Beijing, China | 1214 | 115.583296 | 39.837607 |
| *C. brevicaudata* | BJFC | LRD 20180702-14 | Baihua mountain, mentougou district, Beijing, China | 1119 | 115.577416 | 39.83787 |
| *C. brevicaudata* | PE | T. F. King 342 | Baihua mountain, mentougou district, Beijing, China | 1215 | 115.583339 | 39.837673 |
| *C. brevicaudata* | PE | D. H. Zhu 0129/0702 | Baihua mountain, mentougou district, Beijing, China | 1040 | 115.573082 | 39.837739 |
| *C. brevicaudata* | PE | LXZ 06/25 | Baihua mountain, mentougou district, Beijing, China | 1217 | 115.583382 | 39.837838 |
| *C. brevicaudata* | PE | Anonymous 351 | Baihuashan niangniang temple, mentougou district, Beijing, China | 650 | 115.646038 | 39.998493 |
| *C. brevicaudata* | PE | L. Q. Li 006 | Baihua mountain, mentougou district, Beijing, China | 1132 | 115.578661 | 39.837393 |
| *C. brevicaudata* | PE | Herbarium 2706 | Sunzhazi, Horngou Gate, Huairou District, Beijing, China | 760 | 116.516916 | 40.950247 |
| *C. brevicaudata* | PE | Anonymous 0621 | Beigou Iron Mine Valley, Bohai Town, Beijing, China | 520 | 116.415558 | 40.448639 |
| *C. brevicaudata* | BJFC | J. H.Shi s200215 | Shentangyu, Huairou District, Beijing, China | 330 | 116.621172 | 40.450179 |
| *C. brevicaudata* | BNU | C. Wang s.n. | Wuling mountain, miyun county, Beijing, China | 1280 | 117.476666 | 40.653333 |
| *C. brevicaudata* | PE | Pinggu Exped. 224 | Nanji mountain, liudian town, pinggu county, Beijing, China | 200 | 116.997088 | 40.266085 |
| *C. brevicaudata* | BJTC | F. L. Pan 162 | Badaling, yanqing district, Beijing, China | 614 | 116.025392 | 40.351241 |
| *C. brevicaudata* | PE | [Anonymous 0218](http://www.cvh.ac.cn/spuser/159796) | Shangfangshan, fangshan district, Beijing, China | 500 | 115.82417 | 39.676149 |
| *C. brevicaudata* | PE | BQ-40 | Dajue temple, haidian district, Beijing, China | 240 | 116.092031 | 40.048395 |
| *C. brevicaudata* | BJFC | LRD0006 | Jiufeng forest park, haidian district, Beijing, China | 370 | 116.084753 | 40.062943 |
| *C. brevicaudata* | BJFC | L. Xie 15-JF-07 | Jiufeng forest park, haidian district, Beijing, China | 477 | 116.088409 | 40.057578 |
| *C. brevicaudata* | BJFC | L. Xie 15-JF-H12 | Jiufeng forest park, haidian district, Beijing, China | 420 | 116.086607 | 40.05541 |
| *C. brevicaudata* | PE | Z. Z. Gong 009 | Xiangshan, haidian district, Beijing, China | 180 | 116.213248 | 39.998944 |
| *C. brevicaudata* | BJFC | LRD0084 | Yunmeng mountain, huairou district, Beijing, China | 600 | 116.684946 | 40.583934 |
| *C. brevicaudata* | PE | Z. T. Wang et al. 288 | Jinshan district, haidian district, Beijing, China | 636 | 116.07796 | 40.05651 |
| *C. brevicaudata* | HIMC | Student science unit s.n. | Jinshan district, haidian district, Beijing, China | 500 | 116.058387 | 39.95466 |
| *C. brevicaudata* | BJFC | LRD20180614-01 | Near the Kowloon driving school in mentougou, Beijing, China | 250 | 116.143798 | 39.972523 |
| *C. brevicaudata* | WUK/PE | W. Y. Hsia 2265 | Baihua mountain, mentougou district, Beijing, China | 750 | 115.624647 | 39.850231 |
| *C. brevicaudata* | PE | W. T. Wang 2376 | Caojia road, miyun district, Beijing, China | 560 | 117.415095 | 40.644753 |
| *C. brevicaudata* | PE | W. T. Wang s.n. | Wuling mountain, miyun district, Beijing, China | 1280 | 117.476666 | 40.653333 |
| *C. brevicaudata* | BJFC | LRD0024 | Jingdong laoquan mountain park, pinggu district, Beijing, China | 298 | 117.127519 | 40.309836 |
| *C. brevicaudata* | BJFC | LRD0025 | Jingdong laoquan mountain park, pinggu district, Beijing, China | 310 | 117.129042 | 40.310932 |
| *C. brevicaudata* | BJFC | LRD0032 | Green garden, pinggu district, Beijing, China | 106 | 117.134153 | 40.284978 |
| *C. brevicaudata* | BJFC | LRD0049 | San yang ancient crater scenic spot, pinggu district, Beijing, China | 277 | 117.134152 | 40.284978 |
| *C. brevicaudata* | BJFC | LRD0063 | Sizuolou conservation area, pinggu district, Beijing, China | 780 | 117.221243 | 40.304839 |
| *C. brevicaudata* | BJFC | LRD0082 | Woguayu village, xiongerzhai village, pinggu district, Beijing, China | 343 | 117.127642 | 40.255975 |
| *C. brevicaudata* | PE | H. F. Chow 42023 | Badachu park, shijingshan,Beijing, China | 320 | 116.184339 | 39.972623 |
| *C. brevicaudata* | PE | Anonymous 735 | West hill, shijingshan district, Beijing, China | 320 | 116.184339 | 39.972623 |
| *C. brevicaudata* | BJFC | YQ-151 | Songshan, yanqing district, Beijing, China | 725 | 115.856558 | 40.522867 |
| *C. brevicaudata* | PE | WFS Exped. 324 | Xishan, Beijing, China | 320 | 116.184339 | 39.972623 |
| *C. brevicaudata* | PE | B. Liou 317 | Xiaoxishan national forest park, Beijing, China | 280 | 110.185315 | 39.976647 |
| *C. brevicaudata* | PE | T. P. Wang 5221 | Lintan county, Gansu province, China | 2800 | 103.347411 | 34.594438 |
| *C. brevicaudata* | PE | K. T. Fu 1194 | Wangertang, xiahe county, Gansu province, China | 3100 | 102.771758 | 35.253835 |
| *C. brevicaudata* | PE | W. Y. Hsia 8521 | Boyu, zhuoni county, gansu province, China | 2600 | 103.543594 | 34.556559 |
| *C. brevicaudata* | WUK/IBSC | Huanghe Exped. 655 | Near taibai town, heshui city, gansu province, China | 1190 | 108.677428 | 36.111546 |
| *C. brevicaudata* | WUK | Y. Q. He 4960 | Zhulin ditch, liancheng town, yongdeng county, gansu province, China | 2050 | 103.264287 | 36.739316 |
| *C. brevicaudata* | NAS | J. F. Rock 12750 | Lianhua mountain, Gansu province, China | 3000 | 103.760664 | 34.94823 |
| *C. brevicaudata* | PE/NAS | Huanghe Exped. 3703 | Tumen pass, linxia county, Gansu province, China | 2160 | 103.468156 | 35.479076 |
| *C. brevicaudata* | PE | Huanghe Exped.1877 | Kongtong mountain, Gansu province, China | 1655 | 106.529668 | 35.557634 |
| *C. brevicaudata* | PE | Z. W. Zhang 253 | Baiyanglin to gongchagou, Gansu province, China | 2000 | 105.233474 | 34.392876 |
| *C. brevicaudata* | WUK | Loe - Plat. Exped 4798 | Tulugou valley, liancheng town, Gansu province, China | 2300 | 102.752587 | 36.659673 |
| *C. brevicaudata* | PE | Huanghe Exped. 5966 | The slopes of xinglong mountain, Gansu province, China | 2380 | 104.050203 | 35.77941 |
| *C. brevicaudata* | PE | Chengde Exped. 1775 | Dazhangzi, Hebei province, China | 195 | 118.959497 | 40.400813 |
| *C. brevicaudata* | IFP | W. Wang 3012 | Chengde city, Hebei province, China | 430 | 117.975574 | 40.955917 |
| *C. brevicaudata* | PE/WUK | K. M. Liou 3296 | Longquan village, longquan pass town, Hebei province, China | 930 | 113.852683 | 38.920094 |
| *C. brevicaudata* | PE/HNWP | Hebei Exped. 2977 | Dianziliang, Hebei province, China | 1700 | 114.544553 | 39.568335 |
| *C. brevicaudata* | PE | Anonymous 2057 | Xiaowutai mountain, Hebei province, China | 1500 | 114.920095 | 39.859017 |
| *C. brevicaudata* | WUK | H. W. Kung 982 | Xiaowutai mountain, Hebei province, China | 1520 | 114.920086 | 39.859023 |
| *C. brevicaudata* | PE | H. F. Chow 43488 | Xingtai city, Hebei province, China | 550 | 114.104498 | 36.960181 |
| *C. brevicaudata* | HNWP | X. L. Huang 3350 | Nantai village, Hebei province, China | 800 | 115.207632 | 39.549479 |
| *C. brevicaudata* | PE | S. Y. Qin 32 | Yixian, Hebei province, China | 420 | 115.008775 | 39.196228 |
| *C. brevicaudata* | PE | X. L. Huang 4498 | Longguan town, chicheng county, Hebei province, China | 1300 | 115.610276 | 40.773135 |
| *C. brevicaudata* | HNWP/PE | X. L. Huang 4843 | Taiping mountain, Hebei province, China | 1000 | 114.899157 | 40.841768 |
| *C. brevicaudata* | WUK | W. Y. Hsia 2484 | Lingshan, zhuolu county, hebei province, China | 1620 | 115.285134 | 40.021403 |
| *C. brevicaudata* | PE | X. L. Huang 2164 | Laogou village, hebei province, China | 1325 | 115.102502 | 39.882091 |
| *C. brevicaudata* | TIE | Licent 9081 | Yangjiaping, Hebei province, China | 900 | 115.400865 | 39.979906 |
| *C. brevicaudata* | WUK | K. T. Fu 19080 | Slate rock in Lin county, linzhou city, Henan province, China | 700 | 113.722314 | 36.161632 |
| *C. brevicaudata* | WUK | Loe. - Plat. Exped | Shaoshan, mianchi county, Henan province, China | 1300 | 111.807943 | 34.884181 |
| *C. brevicaudata* | IPF | [B. W. Skvortzov](http://www.cvh.ac.cn/spuser/333636) s.n. | Harbin city, Heilongjiang province, China | 160 | 126.625023 | 45.722185 |
| *C. brevicaudata* | PE | W. T. Wang 98-1 | Ning 'an, Harbin city, Heilongjiang province, China | 550 | 129.340604 | 44.419637 |
| *C. brevicaudata* | NAS | Q. T. Li et al. 55 | Maoer mountain scenic area, harbin city, Heilongjiang province, China | 400 | 127.544573 | 45.290756 |
| *C. brevicaudata* | PE | Y. L. Zhang 1907 | Tuchengzi, harbin city, Heilongjiang province, China | 280 | 129.582604 | 46.0258 |
| *C. brevicaudata* | PE | Y. L. Zhang 1047 | Laoye mountain, jiaohe county, Jilin province, China | 650 | 127.172448 | 43.918283 |
| *C. brevicaudata* | PE | Yanbian Exped. 59-721 | Helong city, Jilin province, China | 700 | 129.046729 | 42.541183 |
| *C. brevicaudata* | PE | Yanbian Exped. 2 groups 721 | Chongshan town, helong city, jilin city, Jilin province, China | 900 | 129.05285 | 42.549569 |
| *C. brevicaudata* | NAS | Y. Yabe s.n. | Dongling park, Liaoning province, China | 91 | 123.58535 | 41.836562 |
| *C. brevicaudata* | PE | Sato 7587 | Fengtian dongling park, Liaoning Province, China | 90 | 123.58539 | 41.836567 |
| *C. brevicaudata* | G/K/LE | Przewalski s.n. | Alxa League, Inner Mongolia autonomous region, China | 2350 | 105.868716 | 38.883803 |
| *C. brevicaudata* | WUK | Y. Y. Pai 96 | Helan mountain, Inner Mongolia autonomous region, China | 2300 | 105.891093 | 38.953242 |
| *C. brevicaudata* | HIMC | Ximeng Exped. 298 | Keshiketeng banner, Inner Mongolia autonomous region, China | 1700 | 117.40547 | 43.486734 |
| *C. brevicaudata* | HIMC | W. S. Yang 991 | Yihegong township, Inner Mongolia autonomous region, China | 1350 | 118.297163 | 42.826179 |
| *C. brevicaudata* | WUK | T.P. Wang 2405 | Daqingshan nature reserve, Inner Mongolia autonomous region, China | 1800 | 111.4993 | 40.924007 |
| *C. brevicaudata* | WUK | W. Y. Hsia 3065 | Wula mountain, Inner Mongolia autonomous region, China | 1510 | 108.899632 | 40.726658 |
| *C. brevicaudata* | WUK | Loe. - Plat. Exped. 4160 | Hanman mountain, Inner Mongolia autonomous region, China | 1500 | 112.300991 | 40.652451 |
| *C. brevicaudata* | HIMC | Y. C. Ma 265 | Liangshan, zhuozi county, Inner Mongolia autonomous region, China | 1503 | 114.025039 | 40.946508 |
| *C. brevicaudata* | HIMC | Nei Mongol Exped 74-68 | Duolun county, Inner Mongolia autonomous region, China | 1450 | 117.143369 | 42.099407 |
| *C. brevicaudata* | WUK | J. X. Yang 5548 | Yunfog mountain, Ningxia hui autonomous region, China | 2000 | 106.389619 | 36.242469 |
| *C. brevicaudata* | SYS | R. C. Ching 1074 | Helan mountain, Ningxia hui autonomous region, China | 1750 | 106.012836 | 38.745716 |
| *C. brevicaudata* | WUK | Loe. - Plat. Exped. 3998 | Luoshan, Ningxia hui autonomous region, China | 2260 | 106.283259 | 37.343736 |
| *C. brevicaudata* | WUK | K. M. Liou 5955 | Datong hui tu autonomous county, Qinghai province, China | 2530 | 101.097235 | 36.935696 |
| *C. brevicaudata* | HNWP | S. W. Liou 2955 | Tang quan, qili temple, gushan town, Qinghai province, China | 2800 | 102.71141 | 36.054569 |
| *C. brevicaudata* | G/LE | Przewalski s.n. | Guide county, Qinghai province, China | 3500 | 101.611228 | 35.97388 |
| *C. brevicaudata* | WUK | Loe. - Plat. Exped. 1883 | Big magnetic kiln, Shanxi Province, China | 1500 | 113.738533 | 39.622184 |
| *C. brevicaudata* | WUK | Loe. - Plat. Exped. 3593 | Boqiang village, Shanxi Province, China | 1800 | 113.633213 | 39.141583 |
| *C. brevicaudata* | WUK/IBSC | T.P. Wang 3722 | Xi county, linfen city, Shanxi Province, China | 1400 | 111.232086 | 36.715569 |
| *C. brevicaudata* | PE/HNWP | X. Y. Liu 20495 | Ganquan village, linfen city, Shanxi Province, China | 1800 | 111.850232 | 35.343278 |
| *C. brevicaudata* | K | T. P. Wang 2782 | Fenyang city, Shanxi Province, China | 1000 | 111.621785 | 37.362374 |
| *C. brevicaudata* | HNWP | K. C. Kuan &Y. L. Chen 2065 | Wutai mountain, xinzhou city, Shanxi Province, China | 1560 | 113.457778 | 38.882243 |
| *C. brevicaudata* | PE/WUK | Huanghe Exped. 56-6825 | Huaqingchi, xi 'an city, Shaanxi province, China | 600 | 109.209585 | 34.361874 |
| *C. brevicaudata* | WUK | Y. L. Xiao 905 | Daxigou, changjiao village, xianyang city, Shaanxi province, China | 1340 | 108.694621 | 35.328036 |
| *C. brevicaudata* | WUK | Loe. - Plat. Exped. 532 | Shangzhen forest park, yan 'an city, Shaanxi province, China | 1300 | 108.731187 | 35.636993 |
| *C. brevicaudata* | WUK | J. X. Yang 4662 | Nanniwan, yan 'an city, Shaanxi province, China | 1200 | 109.665854 | 36.315918 |
| *C. brevicaudata* | PE/WUK/IBS | K. T. Fu 7793 | He jiagou, yan 'an city, Shaanxi province, China | 900 | 110.181066 | 36.939084 |
| *C. brevicaudata* | PE | Shengan Exped. 10822 | Haojiagou, yan 'an city, Shaanxi province, China | 1280 | 108.890437 | 36.920029 |
| *C. brevicaudata* | PE/WUK | Huanghe Exped. 56-7570 | Shiwan town, yulin city, Shaanxi province, China | 1150 | 109.437823 | 37.478991 |
| *C. brevicaudata* | PE/WUK | Huanghe Exped. 56-7737 | Chief ditch of jingbian county, yulin city, Shaanxi province, China | 1620 | 108.677377 | 37.54527 |
| *C. brevicaudata* | PE/IBSC/WUK | K. T. Fu 7902 | Sanshilipu, yulin city, Shaanxi province, China | 1350 | 109.381211 | 37.121333 |
| *C. brevicaudata* | PE | X. Li 78267 | Jinchuan county, Sichuan province, China | 2870 | 102.054742 | 31.457347 |
| *C. brevicaudata* | PE | W. L. Chen et al. 8565 | Shuzhenggou, Sichuan province, China | 2300 | 103.92056 | 33.244169 |
| *C. brevicaudata* | GH/UPS | H. Smith 4577 | Songpan county, Sichuan province, China | 3200 | 103.571995 | 32.644914 |
| *C. brevicaudata* | PE | X. S. Zhang 6997 | Xiaojin county, Sichuan province, China | 2900 | 102.357211 | 30.989347 |
| *C. brevicaudata* | PE/WUK/NAS | X. Li 71883 | Dang ba, malkang county, Sichuan province, China | 2340 | 102.039401 | 31.653876 |
| *C. brevicaudata* | PE/CDBI | Sichuan Exped. 4340 | Center rong township, Sichuan province, China | 2760 | 99.160649 | 29.198726 |
| *C. brevicaudata* | PE | Sichuan Exped. 4341 | Daocheng county, Sichuan province, China | 2900 | 100.332615 | 29.038833 |
| *C. brevicaudata* | PE/WUK/HGAS | W. K. Hu 11271 | Run horse mountain, Sichuan province, China | 2700 | 101.962459 | 30.043327 |
| *C. brevicaudata* | PE | T. T. Yu 13317 | Ganzi Tibetan autonomous prefecture, Sichuan province, China | 3200 | 99.817257 | 28.93473 |
| *C. brevicaudata* | PE/WUK/NAS | X. Li 73852 | Luhua town, heishui county, Sichuan province, China | 2450 | 102.985814 | 32.063118 |
| *C. brevicaudata* | PE | F. T. Wang 1509 | Mao county, Sichuan province, China | 2000 | 103.86915 | 31.678271 |
| *C. brevicaudata* | PE | P. K. Hsiao & T. K. Mi 20305 | Ruoergai, Sichuan province, China | 3445 | 102.870559 | 33.488729 |
| *C. brevicaudata* | BNU | S. Y. He 17407 | Panshan mountain, jixian county, Tianjin, China | 300 | 117.342503 | 40.08772 |
| *C. brevicaudata* | PE | Qingzang Exped. 10952 | Zha, qaru county, Tibet autonomous region, China | 2500 | 98.440063 | 28.445435 |
| *C. brevicaudata* | PE/IBSC | T. T. Yu 10175 | Deqin county, Yunnan province, China | 3240 | 98.918337 | 28.845211 |
| *C. heracleifolia* | PE | K. C. Kuan et al. 12 | Atoll acetabular valley, changping district, Beijing, China | 320 | 116.180882 | 40.341319 |
| *C. heracleifolia* | PE | H. F. Chow 41588 | Nankou, changping district, Beijing, China | 120 | 116.137857 | 40.243142 |
| *C. heracleifolia* | PE | PE Exped. 56-1518 | Xiakou, changping district, Beijing, China | 400 | 116.241409 | 40.338222 |
| *C. heracleifolia* | PE | Changping Exped. 12 | Mountain gully, baihe village, changping district, Beijing, China | 450 | 116.393949 | 40.325091 |
| *C. heracleifolia* | PE | H. F. Chow 41707 | Shangfangshan, fangshan district, Beijing, China | 500 | 115.82417 | 39.676149 |
| *C. heracleifolia* | GH | Read 727 | Great jue temple, haidian district, Beijing, China | 240 | 116.092031 | 40.048395 |
| *C. heracleifolia* | BJFC | LRD002 | JiuFeng national forest park, haidina district,Beijing, China | 370 | 116.084753 | 40.062943 |
| *C. heracleifolia* | PE | PE Exped. 56-2771 | Miaofeng mountain, haidian district, Beijing, China | 260 | 116.060454 | 39.964898 |
| *C. heracleifolia* | BNU/GH | Cowdry 1009 | Wofo temple, haidian district, Beijing, China | 200 | 116.20678 | 40.008196 |
| *C. heracleifolia* | BJFC | LRD20180702-01 | Xiangshan, haidian district, Beijing, China | 180 | 116.213248 | 39.998944 |
| *C. heracleifolia* | PE | W. T. Wang 03-1 | Xiangshan botanical garden, haidian district, Beijing, China | 200 | 116.184216 | 39.988175 |
| *C. heracleifolia* | PE | W. T. Wang 0602 | The Summer Palace in haidian district, Beijing, China | 75 | 116.266123 | 39.999169 |
| *C. heracleifolia* | PE | D. H. Zhu 0121 | Huangantuo village, baihuashan, mentougou district, Beijing, China | 1072 | 115.594196 | 39.868181 |
| *C. heracleifolia* | PE/S | T. F. King 599 | Baihua mountain, mentougou district, Beijing, China | 1280 | 115.624647 | 39.850231 |
| *C. heracleifolia* | PE | W. T. Wang 3092 | Qianjuntai, mentougou district, Beijing, China | 460 | 115.853395 | 39.932412 |
| *C. heracleifolia* | BNU | Z. Y. Mi s.n. | Xiaolong gate, mentougou district, Beijing, China | 1200 | 115.436657 | 39.968612 |
| *C. heracleifolia* | BJFC | LRD0009 | Jingdong laoquan mountain park, pinggu district, Beijing, China | 300 | 117.127519 | 40.309838 |
| *C. heracleifolia* | BJFC | LRD0030 | Green garden, pinggu district, Beijing, China | 294 | 117.134153 | 40.286925 |
| *C. heracleifolia* | BJFC | LRD0039 | San yang ancient crater scenic spot, pinggu district, Beijing, China | 280 | 117.151937 | 40.294912 |
| *C. heracleifolia* | BJFC | LRD0070 | Woguayu village, xiongerzhai village, pinggu district, Beijing, China | 340 | 117.127642 | 40.255976 |
| *C. heracleifolia* | K/PE | T. N. Liou 1395 | Badachu park, shijingshan,Beijing, China | 320 | 116.184339 | 39.972623 |
| *C. heracleifolia* | PE | Z. Y. Cao 92 | Badaling forest park, yanqing, Beijing, China | 680 | 116.025392 | 40.351241 |
| *C. heracleifolia* | PE | D. Z. Fu & Q. Y. Xiang 163 | Songshan, yanqing district, Beijing, China | 850 | 115.856558 | 40.522867 |
| *C. heracleifolia* | BNU | S. Y. He & R. T. Yin 21138 | Handan city, Hebei province, China | 550 | 114.009766 | 36.82398 |
| *C. heracleifolia* | PE | S. Shi & W. D. Liang SZ4188 | Wuyue village, lingshou county, Hebei province, China | 800 | 113.891717 | 38.679235 |
| *C. heracleifolia* | PE | Y. Liu 11893 | Dongling, zunhua city, tangshan city, Hebei province, China | 225 | 117.637534 | 40.193291 |
| *C. heracleifolia* | PE | Y. Liu 11349 | Xiao wutai mountain in yu county, Hebei province, China | 1420 | 114.920095 | 39.859017 |
| *C. heracleifolia* | BNU | S. Y. He 2289 | Qiu county, xingtai city, Hebei province, China | 500 | 114.251396 | 37.3559 |
| *C. heracleifolia* | PE | H. F. Chow 43430 | Southwest of xingtai county, Hebei province, China | 550 | 114.104498 | 36.960181 |
| *C. heracleifolia* | PE/LE | T. N. Liou & P. Y. Fu 4594 | Wuling mountain, xinglong county, Hebei province, China | 830 | 117.503806 | 40.637302 |
| *C. heracleifolia* | PE | X. L. Huang et al. 868 | Yi county, Hebei province, China | 330 | 115.406859 | 39.442381 |
| *C. heracleifolia* | GH | H. Smith 312 | Xilingshan, yangjiaping,zhuolou country, Hebei province, , China | 1250 | 115.377487 | 40.062195 |
| *C. heracleifolia* | PE | Henan Exped. 59-50705 | Dengfeng city, Henan province, China | 920 | 113.045809 | 34.48487 |
| *C. heracleifolia* | JJF | C. M. Tan & G. H. Yi 10595 | Songshan shaoshi mountain, dengfeng city, Henan province, China | 730 | 112.991633 | 34.508035 |
| *C. heracleifolia* | PE | Yuntaishan Exped. 0095 | Yuntai mountain, xiuwu county, Henan province, China | 370 | 113.394261 | 35.423634 |
| *C. heracleifolia* | PE | Henan Exped. 59-55011 | Yuzhou city, xuchang city, Henan province, China | 460 | 113.502724 | 34.339879 |
| *C. heracleifolia* | NAS | 07 grade 2 groups 07-2-025 | Huaguo mountain, lianyungang city, Jiangsu province, China | 220 | 119.266661 | 34.635723 |
| *C. heracleifolia* | IBSC | T. N. Liou 6821 | Honggu 'an, qianshan district, Liaoning province, China | 120 | 122.923861 | 41.055015 |
| *C. heracleifolia* | IBSC/LE/NAS/PE | Y. L. Zhou et al. 2542 | Nangou, qianshan district, Liaoning province, China | 230 | 123.030888 | 41.003333 |
| *C. heracleifolia* | PE | Y. Yabe s.n. | Caohekou, benxi city, Liaoning province, China | 400 | 123.919416 | 40.877284 |
| *C. heracleifolia* | IFP | T. N. Liou 359 | Dongling park, shenyang city, Liaoning province, China | 90 | 123.58535 | 41.836562 |
| *C. heracleifolia* | PE | Y. L. Zhou et al. 2650 | Xiong yuecheng, yingkou city, Liaoning province, China | 340 | 122.233325 | 40.151111 |
| *C. heracleifolia* | HIMC | Y. M. Zhu 421 | Daheishan, chifeng city, Inner Mongolia autonomous region, China | 820 | 119.979745 | 41.900583 |
| *C. heracleifolia* | GH/K/NAS | C. Y. Chiao 3073 | Longdong, jinan city, Shandong province, China | 450 | 117.116805 | 36.597278 |
| *C. heracleifolia* | PE | C. Y. Guo 55187-10 | Changqing district, jinan city, Shandong province, China | 130 | 116.771218 | 36.54536 |
| *C. heracleifolia* | PE | Y. T. Hou et al. 98001-1 | Liujiazhai reservoir, linyi city, Shandong province, China | 600 | 117.924336 | 35.476821 |
| *C. heracleifolia* | QFNU | 09 groups 201609062 | Mengshan north mountain stream, linyi city, shandong province, China | 210 | 117.526016 | 35.525365 |
| *C. heracleifolia* | NAS PE | T. Y. Chou et al. 7182 | Taishan scenic area, Shandong province, China | 350 | 117.100439 | 36.217841 |
| *C. heracleifolia* | PE | C. H. Duan 00405 | West yankou farm yard, Shanxi Province, China | 1640 | 113.628501 | 39.214067 |
| *C. heracleifolia* | GH PE | T. Tang 1526 | Jiaocheng county, Shanxi Province, China | 900 | 112.097265 | 37.54532 |
| *C. heracleifolia* | PE | Licent 11366 | Licheng county, Shanxi Province, China | 970 | 113.451524 | 36.50018 |
| *C. heracleifolia* | HNWP | S. Y. Bao 962 | Houping village, ruicheng county, Shanxi Province, China | 1150 | 110.557191 | 34.773499 |
| *C. heracleifolia* | PE | K. C. Kuan & Y. L. Chen 2675 | Wutai mountain, xinzhou city, Shanxi Province, China | 1740 | 113.596666 | 39.00821 |
| *C. heracleifolia* | PE/NAS/IBK | Huanghe Exped. 57-582 | Snowflake mountain, Shanxi Province, China | 1600 | 110.514101 | 34.809575 |
| *C. heracleifolia* | PE/HNWP | S. Y. Bao 744 | Liishan town, yuncheng city, Shanxi Province, China | 510 | 111.895816 | 35.268316 |
| *C. heracleifolia* | K | Yinger et al. 2849 | Baengnyeong-ro, Korea | 43 | 124.698772 | 37.963147 |
| *C. tubulosa* | KYO | Koidzumi s.n. | Korea, Hamgyong-Namdo, North Korea | 340 | 128.303213 | 40.375854 |
| *C. tubulosa* | K | Erskine & Cowley 166 | Kangwon-do: Mt. Odae National Park Beyer, Korea | 775 | 128.632954 | 37.797173 |
| *C. tubulosa* | KYO | Koidzumi s.n. | Kyongyi-do, Korea | 270 | 127.499682 | 37.425059 |
| *C. tubulosa* | PE | Changping Exped. 41 | Baihe village, changping district, Beijing, China | 410 | 116.394436 | 40.326843 |
| *C. tubulosa* | PE | H. F. Chow 40471/40513 | Nankou, changping district, Beijing, China | 125 | 116.137857 | 40.243142 |
| *C. tubulosa* | PE | K. M. Liou 666 | Ming tombs in changping district, Beijing, China | 140 | 116.251607 | 40.251672 |
| *C. tubulosa* | PE | J. Zhang 2023 | Xiakou village, changping district, Beijing, China | 360 | 116.242883 | 40.34491 |
| *C. tubulosa* | PE | W. Y. Xia 3217a | Shangfangshan, fangshan district, Beijing, China | 500 | 115.82417 | 39.676149 |
| *C. tubulosa* | PE | T. N. Liou 1399 | Beijing zoo, haidian district, Beijing, China | 55 | 116.326691 | 39.941298 |
| *C. tubulosa* | PE | Anonymous 8 | Dajue temple, haidian district, Beijing, China | 196 | 116.092031 | 40.048395 |
| *C. tubulosa* | BJFC | L. Xie 2015-JF-H02/03/04/06/08/09/10/11 | Jiufeng national park, haidian district, Beijing, China | 718 | 116.082487 | 40.061881 |
| *C. tubulosa* | BJFC | LRD0002 | Jiufeng national park, haidian district, Beijing, China | 715 | 116.082745 | 40.061782 |
| *C. tubulosa* | BNU | S. Y. He s.n. | Jinshan district, haidian district, Beijing, China | 500 | 116.058387 | 39.95466 |
| *C. tubulosa* | PE | Z. T. Wang 156 | Qinglongqiao, haidian district, Beijing, China | 65 | 116.26456 | 39.999578 |
| *C. tubulosa* | BNU | Cowdry 41 | Wofo temple, haidian district, Beijing, China | 200 | 116.20678 | 40.008196 |
| *C. tubulosa* | GH | Bartholomev & Boufford 2038 | Xiangshan, haidian district, Beijing, China | 180 | 116.213248 | 39.998944 |
| *C. tubulosa* | PE | W. T. Wang 0601 | The Summer Palace, haidian district, Beijing, China | 75 | 116.266123 | 39.999169 |
| *C. tubulosa* | BNU | S. Y. He 33088 | Yunmeng mountain, huairou district, Beijing, China | 600 | 116.684946 | 40.583934 |
| *C. tubulosa* | BJFC | LRD 20180702-15/16/17/18, LRD0094-0102 | Baihua mountain, mentougou district, Beijing, China | 1124 | 115.577545 | 39.837805 |
| *C. tubulosa* | PE | W. Y. Xia 2005 | Baihua mountain, mentougou district, Beijing, China | 700 | 115.624647 | 39.850231 |
| *C. tubulosa* | PE | T. F. King 417 | Baihua mountain, mentougou district, Beijing, China | 1173 | 115.58115 | 39.840111 |
| *C. tubulosa* | BNU | BNU 1140 | Baihua mountain, mentougou district, Beijing, China | 1186 | 115.581965 | 39.838562 |
| *C. tubulosa* | PE | C. W. Wang 60292 | Baihua mountain, mentougou district, Beijing, China | 1206 | 115.58291 | 39.837541 |
| *C. tubulosa* | PE | W. T. Wang 574 | Huanantuo village, Baihuashan, mentougou district, Beijing, China | 1102 | 115.597801 | 39.869037 |
| *C. tubulosa* | GH | Bartholomev & Boufford 2060 | Jietai temple, mentougou district, Beijing, China | 390 | 116.083978 | 39.869142 |
| *C. tubulosa* | K | Hancock s.n. | Jiulonggou, mentougou district, Beijing, China | 600 | 115.817803 | 39.977732 |
| *C. tubulosa* | BJFC | LRD0104 | Xiaolong gate, mentougou district, Beijing, China | 1007 | 115.436657 | 39.968612 |
| *C. tubulosa* | BJFC | LRD0015/0021/0022 | Jingdong laoquan mountain park, pinggu district, Beijing, China | 301 | 117.128205 | 40.311767 |
| *C. tubulosa* | BNU | S. Y. He 13589 | Wuling mountain, miyun county, Beijing, China | 1280 | 117.476666 | 40.653333 |
| *C. tubulosa* | BJFC | LRD0058/0059/0060/0061/0062 | Sizuolou conservation area, pinggu district, Beijing, China | 650 | 117.221245 | 40.304839 |
| *C. tubulosa* | BJFC | LRD0075/0077 | Woguayu village, xiongerzhai village, pinggu district, Beijing, China | 256 | 117.130351 | 40.256276 |
| *C. tubulosa* | PE | H. F. Chow 42046 | Badachu park, shijingshan,Beijing, China | 650 | 116.184339 | 39.972623 |
| *C. tubulosa* | PE | D. Z. Fu & Q. Y. Xiang 83012 | Songshan, yanqing county, Beijing, China | 850 | 115.856558 | 40.522867 |
| *C. tubulosa* | PE | W. Y. Hsia 2582 | Baihuashan, Hebei province, China | 1833 | 115.578661 | 39.815887 |
| *C. tubulosa* | BNU | J. C. Liu 1161 | Yi county, baoding city, Hebei province, China | 330 | 115.406859 | 39.442381 |
| *C. tubulosa* | PE | W. Y. Hsia 1944 | Changli county, Hebei province, China | 300 | 119.127307 | 39.757931 |
| *C. tubulosa* | BNU | Z. T. Yin 19898 | Fengning manchu autonomous county, Hebei province, China | 780 | 116.629983 | 41.198809 |
| *C. tubulosa* | BNU | S. Y. He 20318 | Baishishan, laiyuuan county, Hebei province, China | 995 | 114.061485 | 39.317651 |
| *C. tubulosa* | PE | Anonymous s. n. | Qinhuangdao qinglong manchu autonomous county, Hebei province, China | 380 | 118.973903 | 40.399916 |
| *C. tubulosa* | P | Licent 1580 | Shanhaiguan district, qinhuangdao city, Hebei province, China | 200 | 119.717492 | 40.042176 |
| *C. tubulosa* | P | X. L. Huang et al. 2357/5622/6041 | Xiao wutai mountain, yu county, Hebei province, China | 1238 | 114.920095 | 39.859017 |
| *C. tubulosa* | PE | T. N. Liou 4604 | Hongmei temple to the bell drum courtyard, xinglong county, Hebei province | 800 | 117.51182 | 40.51084 |
| *C. tubulosa* | PE/K | Licent 2494 | Huailai county, zhangjiakou city, Hebei province, China | 1000 | 115.623469 | 40.415857 |
| *C. tubulosa* | PE/S | T. F. King 237 | Xiling mountain, zhuolu county, Hebei province, China | 1250 | 115.377487 | 40.062195 |
| *C. tubulosa* | GH/UPS | H. Smith 291/302 | Yangjiaping, zhuolu country, Hebei province, China | 900 | 115.400865 | 39.979906 |
| *C. tubulosa* | PE | K. M. Liou 425 | Dongling, zunhua city, Hebei province, China | 250 | 117.638979 | 40.194668 |
| *C. tubulosa* | HHBG | Anonymous 20647 | Under 9th floor of yuntai mountain, Jiangsu province, China | 241 | 119.448745 | 34.707399 |
| *C. tubulosa* | IBSC | T. Y. Chou et al.21242 | Guanshan, ganyu district, Jiangsu province, China | 230 | 118.954727 | 35.003184 |
| *C. tubulosa* | NAS | T. Y. Chou et al. 20909 | Yuntai mountain, lianyungang city, Jiangsu province, China | 150 | 119.319251 | 34.682076 |
| *C. tubulosa* | IFP | S. X. Li 5916 | Qianshan, anshan district, Liaoning province, China | 100 | 122.927536 | 41.059298 |
| *C. tubulosa* | IFP | Z. S. Qin et al. 531 | Chaoyang beigou, wulv mountain national forest, Liaoning province, China | 159 | 121.713398 | 41.603479 |
| *C. tubulosa* | PE/WUK/IBKI | C. S. Wang 4054 | Naozhi ditch, huanren manchu autonomous county, benxi city, Liaoning province | 410 | 125.427681 | 41.053451 |
| *C. tubulosa* | IFP | C. S. Wang 3705 | Left wing Mongolian autonomous county, Liaoning province, China | 375 | 119.729902 | 41.144682 |
| *C. tubulosa* | IFP | Z. Wang et al. 889 | Dalian, liaoning province, China | 140 | 121.609373 | 38.884467 |
| *C. tubulosa* | IFP | J. Wei 38 | Pine tree town, dalian city, Liaoning province, China | 170 | 122.121998 | 39.811873 |
| *C. tubulosa* | IFP | W. Wang 1286 | Dalu island, Liaoning province, China | 110 | 123.737104 | 39.75821 |
| *C. tubulosa* | IFP | W. C. Jiang 68 | Fengcheng manchu autonomous county, Liaoning province, China | 300 | 124.098511 | 40.427541 |
| *C. tubulosa* | IFP | W. Wang 1047 | Yuanbao mountain, dandong city, Liaoning province, China | 110 | 124.391866 | 40.150729 |
| *C. tubulosa* | IFP | S. Z. Liou 133 | Bahushan forest farm, faku county, Liaoning province, China | 230 | 123.199561 | 42.474551 |
| *C. tubulosa* | IFP | C. S. Wang 3221 | Jianchang county, huludao city, Liaoning province, China | 530 | 119.88206 | 40.799781 |
| *C. tubulosa* | IFP | S. X. Li 542 | Suizhong county, huludao city, Liaoning province, China | 175 | 120.206969 | 40.327985 |
| *C. tubulosa* | PE | Z. Wang 3544 | Jinzhou city, Liaoning province, China | 140 | 121.139407 | 41.064094 |
| *C. tubulosa* | PE/SYS | W. Wang 334 | Matou mountain, lingyuan city, Liaoning province, China | 435 | 119.237284 | 40.728984 |
| *C. tubulosa* | GH | Komarov 708 | Dongling park, shenyang city, Liaoning province, China | 90 | 123.58535 | 41.836562 |
| *C. tubulosa* | PE | B. Z. Guo 640 | Tieling county, tieling city, Liaoning province | 150 | 123.76648 | 42.20813 |
| *C. tubulosa* | PE | T. Y. Chou et al. 1226 | Chaoyin waterfall, Qingdao city, Shandong province, China | 725 | 120.614004 | 36.193066 |
| *C. tubulosa* | CAF | Z. X. Zhao 16128 | Laishan town, yantai city, Shandong province, China | 200 | 121.408251 | 37.376457 |
| *C. tubulosa* | PE | T. N. Liou & K. M. Liou 1391/1485 | Muping kunyu mountain, Shandong province, China | 380 | 121.748696 | 37.275959 |
| *C. tubulosa* | PE | PE Exped. 56-2002 | Panshan mountain, jixian county, Tianjin, China | 300 | 117.342503 | 40.08772 |
| *C. pinnata* | BNU | S. Y. He 15037 | Baihua mountain, mentougou district, Beijing, China | 750 | 115.624647 | 39.850231 |
| *C. pinnata* | PE | B. Liou 330 | Xiaoxishan national forest park, mentougou district, Beijing, China | 330 | 116.188595 | 39.978888 |
| *C. pinnata* | PE | S. Y. He 75037 | baihuashan forest farm, mentougou district, Beijing, China | 1200 | 115.570928 | 39.830436 |
| *C. pinnata* | BJFC | L. Xie 20120720 | Sunzhazi, Horngou Gate, Huairou District, Beijing, China | 760 | 116.516916 | 40.950247 |
| *C. pinnata* | PE | Anonymous 0621 | Beigou, iron yuyu, bohai town, huairou district, Beijing, China | 520 | 116.415558 | 40.448639 |
| *C. pinnata* | PE | s200218 | Shentang valley, huairou district, Beijing, China | 330 | 116.621172 | 40.450179 |
| *C. pinnata* | BNU | C. Wang s.n. | Wuling mountain, miyun district, Beijing, China | 1280 | 117.476666 | 40.653333 |
| *C. pinnata* | PE | Pinggu Exped. 224 | Nanji mountain, liudian town, pinggu district, Beijing, China | 200 | 116.997088 | 40.266085 |
| *C. pinnata* | BNU | S. Y. He s.n. | Nanshan village, pinggu county, Beijing, China | 170 | 117.270119 | 40.113907 |
| *C. pinnata* | BJFC | L. Xie s.n. | Badaling National Forest Park, Beijing, China | 614 | 116.025392 | 40.351241 |
| *C. pinnata* | BJFC | L. Xie s.n. | Shangfangshan, fangshan district, Beijing, China | 500 | 115.82417 | 39.676149 |
| *C. pinnata* | BJFC | L. Xie 20130821 | Dajue temple, haidian district, Beijing, China | 240 | 116.092031 | 40.048395 |
| *C. pinnata* | BJFC | LRD0005 | Wangjing Tower, jiufeng national forest park, haiidan district, Beijing, China | 700 | 116.069281 | 40.062943 |
| *C. pinnata* | BJFC | LRD0083 | Yunmeng mountain, huairou district, Beijing, China | 1115 | 116.684946 | 40.583934 |
| *C. pinnata* | HIMC | Student science unit s.n. | Jinshan district, haidian district, Beijing, China | 500 | 116.058387 | 39.95466 |
| *C. pinnata* | BJFC | LRD0026 | Jingdong laoquan mountain park, pinggu district, Beijing, China | 298 | 117.127519 | 40.309836 |
| *C. pinnata* | BJFC | LRD0031 | Green garden, pinggu district, Beijing, China | 106 | 117.134152 | 40.284977 |
| *C. pinnata* | BJFC | LRD0033 | San yang ancient crater scenic spot, pinggu district, Beijing, China | 277 | 117.134153 | 40.284978 |
| *C. pinnata* | BJFC | LRD0053 | Sizuolou conservation area, pinggu district, Beijing, China | 780 | 117.221241 | 40.304838 |
| *C. pinnata* | BJFC | LRD0068 | Woguayu village, xiongerzhai village, pinggu district, Beijing, China | 343 | 117.127642 | 40.255975 |
| *C. pinnata* | PE | Anonymous 735 | West hill, shijingshan district, Beijing, China | 320 | 116.184339 | 39.972623 |
| *C. pinnata* | PE | Licent 9831 | Songshan, yanqing, Beijing, China | 725 | 115.856558 | 40.522867 |
| *C. pinnata* | PE | Anonymous 735 | Xishan, Beijing, China | 320 | 116.184339 | 39.972623 |
| *C. pinnata* | PE | Chengde Exped. 71-1775 | Dazhangzi, Hebei province, China | 195 | 118.959497 | 40.400813 |
| *C. pinnata* | PE | Anonymous 2057 | Xiaowutai mountain, yu county, Hebei province, China | 1500 | 114.920095 | 39.859017 |
| *C. pinnata* | PE | Yi Xian 71-140 | Sanggang village, yi county, Hebei province, China | 420 | 115.008775 | 39.196228 |
| *C. pinnata* | TIE | Licent 9081 | Yangjiaping, zhuolu country, Hebei province, China | 900 | 115.400865 | 39.979906 |
| *C. pinnata* | NAS | Y. Yabe s.n. | Fengtian dongling park, shenyang city, Liaoning Province, China | 91 | 123.58535 | 41.836562 |
| *C. pinnata* | BNU | S. Y. He 17407 | Panshan mountain, jixian county, Tianjin, China, China | 300 | 117.342503 | 40.08772 |
