# Supplementary material for: Natural Hybrid Origin of the Controversial “Species” Clematis × pinnata (Ranunculaceae) Based on Multidisciplinary Evidence
Source: Front Plant Sci. 2021 Oct 12;12:745988. doi: 10.3389/fpls.2021.745988 (PMC8545901; doi:10.3389/fpls.2021.745988)
Supplement: Supplementary Table S6 — The 33 environmental variables used for the niche modeling. [file Table_6.DOCX]

**TABLE S6.** The 33 environmental variables used for the niche modelling.

| Data source | Environmental  variables | Ecological meaning |
| --- | --- | --- |
| WorldClim | BIO1 | Annual Mean Temperature |
|  | **BIO2** | **Mean Diurnal Range (Mean of monthly (max temp-min temp))** |
|  | BIO3 | Isothermality (BIO2/BIO7) (* 100) |
|  | BIO4 | Temperature Seasonality (standard deviation *100) |
|  | BIO5 | Max Temperature of Warmest Month |
|  | BIO6 | Min Temperature of Coldest Month |
|  | BIO7 | Temperature Annual Range (BIO5-BIO6) |
|  | **BIO8** | **Mean Temperature of Wettest Quarter** |
|  | BIO9 | Mean Temperature of Driest Quarter |
|  | BIO10 | Mean Temperature of Warmest Quarter |
|  | BIO11 | Mean Temperature of Coldest Quarter |
|  | BIO12 | Annual Precipitation |
|  | BIO13 | Precipitation of Wettest Month |
|  | BIO14 | Precipitation of Driest Month |
|  | **BIO15** | **Precipitation Seasonality (Coefficient of Variation)** |
|  | BIO16 | Precipitation of Wettest Quarter |
|  | BIO17 | Precipitation of Driest Quarter |
|  | **BIO18** | **Precipitation of Warmest Quarter** |
|  | **BIO19** | **Precipitation of Coldest Quarter** |
| glUV | **UVB1** | **Annual Mean UV-B** |
|  | UVB2 | UV-B Seasonality |
|  | UVB3 | Mean UV-B of Highest Month |
|  | **UVB4** | **Mean UV-B of Lowest Month** |
|  | UVB5 | Sum of Monthly Mean UV-B during Highest Quarter |
|  | UVB6 | Sum of Monthly Mean UV-B during Lowest Quarter |
| FAO | FOR | Forrest land |
|  | GRS | Grass /scrub /woodland |
|  | NVG | Barren /very sparsely vegetated land |
|  | WAT | Mapped water bodies |
|  | GloElev | Global elevation |
| UW | GDD | Growing degree days |
|  | **SpH** | **Soil pH** |
|  | **SOC** | **Soil organic carbon** |

Bold variations are used in MaxEnt modelling.
