# Supplementary material for: Natural Hybrid Origin of the Controversial “Species” Clematis × pinnata (Ranunculaceae) Based on Multidisciplinary Evidence
Source: Front Plant Sci. 2021 Oct 12;12:745988. doi: 10.3389/fpls.2021.745988 (PMC8545901; doi:10.3389/fpls.2021.745988)
Supplement: Supplementary Table S7 — Flow cytometry analysis of Clematis tubulosa and C. pinnata using C. brevicaudata as an external standard reference. [file Table_7.DOC]

**TABLE S7.** Flow cytometry analysis of *C. tubulosa* and *C. pinnata* using *C. brevicaudata* as external standard reference.

| Population | Collection number | Species | G_0_/G_1_ fluorescence value of samples | G_0_/G_1_ fluorescence value of samples compare to external standard each population | The coefficient of variation (CV, %) |
| --- | --- | --- | --- | --- | --- |
| Jiufeng forest park, Haidian district, Beijing (JF) | LRDb11 | *C. brevicaudata* | 21740.78 | / | 6.12 |
| Jiufeng forest park, Haidian district, Beijing (JF) | LRDp1 | *C. pinnata* | 23228.49 | 1.07 | 5.9 |
| Jiufeng forest park, Haidian district, Beijing (JF) | LRDt7 | *C. tubulosa* | 26431.52 | 1.21 | 6.89 |
