# Supplementary material for: Natural Hybrid Origin of the Controversial “Species” Clematis × pinnata (Ranunculaceae) Based on Multidisciplinary Evidence
Source: Front Plant Sci. 2021 Oct 12;12:745988. doi: 10.3389/fpls.2021.745988 (PMC8545901; doi:10.3389/fpls.2021.745988)
Supplement: Supplementary Table S8 — Flow cytometry analysis of Clematis heracleifolia using C. brevicaudata as an external standard reference. [file Table_8.DOCX]

**TABLE S8.** Flow cytometry analysis of *C. heracleifolia* using *C. brevicaudata* as external standard reference.

| Population | Collection number | Species | G_0_/G_1_ fluorescence value of samples | G_0_/G_1_ fluorescence value of samples compare to external standard each population | The coefficient of variation (CV, %) |
| --- | --- | --- | --- | --- | --- |
| Yanqing district, Beijing (YQ) | LRDb9 | *C. brevicaudata* | 18050.07 | / | 5.03 |
| Yanqing district, Beijing (YQ) | LRDh1 | *C. heracleifolia* | 17227.09 | 0.95 | 5.12 |
