# Supplementary material for: Natural Hybrid Origin of the Controversial “Species” Clematis × pinnata (Ranunculaceae) Based on Multidisciplinary Evidence
Source: Front Plant Sci. 2021 Oct 12;12:745988. doi: 10.3389/fpls.2021.745988 (PMC8545901; doi:10.3389/fpls.2021.745988)
Supplement: Supplementary Table S9 — HyDe analysis of Clematis pinnata and its putative parents in each population using the complete SNP data. Sample size of C. pinnata: eight populations, 28 individuals. [file Table_9.DOC]

**TABLE S10.** Percentage contributions and permutation importance of the variables included in the MaxEnt modelling for *Clematis pinnata* and its putative parents.

| Environmental variables | Unit | *C. brevicaudata* (%) | *C. pinnata*  (%) | *C. heracleifolia* (%) | *C. tubulosa*  (%) |
| --- | --- | --- | --- | --- | --- |
| Mean UV-B of Lowest Month (UVB4) | J/m^2^/day | **32.2** | **17.1** | **19.2** | **18.1** |
| Precipitation of Warmest Quarter (BIO18) | mm | **29.7** | **31.7** | **32.2** | **27.3** |
| Precipitation of Coldest Quarter (BIO19) | °C ×10 | **26** | 2.5 | 7.6 | 12.4 |
| Mean Temperature of Wettest Quarter (BIO8) | mm | **8.1** | 2.6 | 2.8 | 2 |
| Mean Diurnal Range (Mean of monthly (max –min) temp) (BIO2) | °C ×10 | 1.4 | 0.2 | 0.6 | 2.2 |
| Precipitation Seasonality (Coefficient of Variation) (BIO15) | mm | 1 | **21** | 6.5 | **18.8** |
| Soil pH (SpH) |  | 0.2 | 5 | **18.5** | 0.4 |
| Annual Mean UV-B (UVB1) | J/m^2^/day | 0.1 | **19.5** | **11.4** | **18.6** |
| Soil organic carbon (SOC) | Kg/m^2^ | 0.1 | 0.1 | 0.5 | 0.2 |
